# Exploiting EST databases for the development and characterization of EST-SSR markers in castor bean (*Ricinus communis *L.)

**DOI:** 10.1186/1471-2229-10-278

**Published:** 2010-12-16

**Authors:** Lijun Qiu, Chun Yang, Bo Tian, Jun-Bo Yang, Aizhong Liu

**Affiliations:** 1 Key Laboratory of Tropical Forest Ecology, Xishuangbanna Tropical Botanical Garden, Chinese Academy of Sciences, 88 Xuefu Road, Kunming 650223, PR China; 2 SW China Germplasm Bank of Wild Species, Kunming Institute of Botany, Chinese Academy of Sciences, Kunming 650204, PR China; 3 Graduate University of Chinese Academy of Sciences, Beijing 100039, PR China

## Abstract

**Background:**

The castor bean *(Ricinus communis *L.), a monotypic species in the spurge family (Euphorbiaceae, 2n = 20), is an important non-edible oilseed crop widely cultivated in tropical, sub-tropical and temperate countries for its high economic value. Because of the high level of ricinoleic acid (over 85%) in its seed oil, the castor bean seed derivatives are often used in aviation oil, lubricants, nylon, dyes, inks, soaps, adhesive and biodiesel. Due to lack of efficient molecular markers, little is known about the population genetic diversity and the genetic relationships among castor bean germplasm. Efficient and robust molecular markers are increasingly needed for breeding and improving varieties in castor bean. The advent of modern genomics has produced large amounts of publicly available DNA sequence data. In particular, expressed sequence tags (ESTs) provide valuable resources to develop gene-associated SSR markers.

**Results:**

In total, 18,928 publicly available non-redundant castor bean EST sequences, representing approximately 17.03 Mb, were evaluated and 7732 SSR sites in 5,122 ESTs were identified by data mining. Castor bean exhibited considerably high frequency of EST-SSRs. We developed and characterized 118 polymorphic EST-SSR markers from 379 primer pairs flanking repeats by screening 24 castor bean samples collected from different countries. A total of 350 alleles were identified from 118 polymorphic SSR loci, ranging from 2-6 per locus (A) with an average of 2.97. The EST-SSR markers developed displayed moderate gene diversity (*H*_e_) with an average of 0.41. Genetic relationships among 24 germplasms were investigated using the genotypes of 350 alleles, showing geographic pattern of genotypes across genetic diversity centers of castor bean.

**Conclusion:**

Castor bean EST sequences exhibited considerably high frequency of SSR sites, and were rich resources for developing EST-SSR markers. These EST-SSR markers would be particularly useful for both genetic mapping and population structure analysis, facilitating breeding and crop improvement of castor bean.

## Background

Castor bean (*Ricinus communis *L., Euphorbiaceae, 2n = 20) is an important non-edible oilseed crop and its seed derivatives are often used in aviation oil, lubricants, nylon, dyes, inks, soaps, adhesive and biodiesel. Among all the vegetable oils, castor bean oil is distinctive due to its high level of ricinoleic acid (over 85%), a fatty acid consisting of 18 carbons, a double bond between C9 and C10, and a hydroxyl group attached to C12. Ricinoleic acid is responsible for castor bean oil interest, with the highest and most stable viscosity index among all the vegetable oils combined with high lubricity, especially under low-temperature conditions. Although it was found that castor bean seeds had been used by people dating from about 4000 BC [1], it is still an unanswered question about the origin of castor bean cultivation. Castor bean's contemporary distribution in the warmer regions is worldwide, although its origin is obscured by wide dissemination in ancient times and the ease and rapidity with which it becomes established. Castor bean is indigenous to southeastern Mediterranean Basin, Eastern Africa, and India, and most probably originated in tropical Africa [2,3]. Because of its high economic value, castor bean is widely cultivated in tropical, sub-tropical and temperate countries, particularly India, China and Brazil [4]. Due to increased demand for castor bean in many countries, breeding and improvement of varieties are drawing great attention from breeders [5].

Although the genus *Ricinus *is considered monotypic, castor bean varies greatly in its growth habit, color of foliage and stems, seed size and oil content [6,7]. Most types are large perennials that often develop into small trees in tropical or subtropical areas; however it is usually shorter and smaller and grown annually in areas prone to frost. It is obvious that castor bean exhibits great phenotypic diversity and phenotypic plasticity to environmental factors. However, little is known about castor bean's genetic diversity and the genetic basis of its phenotypic plasticity. Castor bean is usually considered to be both self- and cross-pollinated by wind, but controlled crossing studies suggest that outcrossing is a frequent mode of reproduction [8,9].

Germplasm collections constitute one of the world's most readily available sources of plant genetic material [10]. The USDA-ARS Plant Genetic Resources Conservation Unit (at Griffin, GA, USA) collected and maintained diverse germplasm resources of castor bean worldwide, which provided valuable germplasms for castor bean breeding and improvement of varieties. There is an increasing need for distinguishing the varieties reliably, establishing their purity, and fingerprinting released varieties, hybrids and the parental lines of castor bean germplasm held in different countries by efficient molecular markers during breeding and improvement of varieties. Most cultivars have low productivity. The castor bean seed, meanwhile, contains the highly toxic protein ricin which seriously limits its usage. The main goal of breeding and improvement of varieties to breeders is to develop high-productivity and nontoxic varieties of castor bean. Developing robust and reliable molecular markers associated with traits of interest will enhance the breeding program efficiency.

Simple sequence repeats (SSRs) or microsatellites showing extensive length polymorphisms have been widely used in DNA fingerprinting, genetic diversity studies, construction of genetic linkage map and breeding applications [11]. Previous studies of genetic diversity suggested that SSRs are more informative and robust than other available molecular marker resources, such as amplified fragment length polymorphism (AFLP) and random amplified polymorphic DNA (RAPD) in castor bean [12,13]. In particular, SSR markers are readily transferable between laboratories as each locus is defined by the primer sequence. SSRs can be used not only for identifying cultivars but also for genetic mapping and marker-assisted selection [14,15]. Development of SSR markers specific to castor bean is critical and should be a priority for assisting in the breeding and improvement of varieties [5]. The SSR markers of castor bean are, however, very limited to date because the *de novo *development of SSRs is a costly and time consuming endeavor [16,17]. The advent of modern genomics age has produced large amounts of publicly available DNA sequence data. In particular, the expressed sequence tags (ESTs) provide a valuable resource for identifying and developing gene-associated SSR markers. Linkage of EST-SSR markers with desired characters may lead to the identification of genes controlling these traits [18]. In addition, EST-SSRs are universal and can be applied in comparative mapping and linkage map construction [19,20]. Therefore, in recent years, EST-SSRs have already been developed for various crops such as wheat and rice [21-25], barley [26-28], grape [29], tomato [30], sugar cane [19], coffee [31-33], oil palm [34] and rubber tree [35].

To our knowledge, there has been no report of development of EST-SSR markers in castor bean to date. Therefore, we report our work on EST-SSRs derived from castor bean ESTs in the National Centre of Bioinformatics Information, USA database, based on (1) the frequency and distribution of SSRs in castor bean ESTs, (2) the establishment and validation of EST-SSR markers for detection of polymorphism in castor bean, and (3) the assessment of genetic relationships among 24 germplasm accessions collected from main diversity centers of castor bean by using EST-SSR markers developed. These rich SSR resources from castor bean EST database are publicly available and the polymorphic EST-SSR markers reported herein would be particularly useful for genetic map-based analyses as well as population genetic studies, facilitating breeding and crop improvement of castor bean.

## Results

### Frequency and distribution of microsatellites

**Figure 1:**
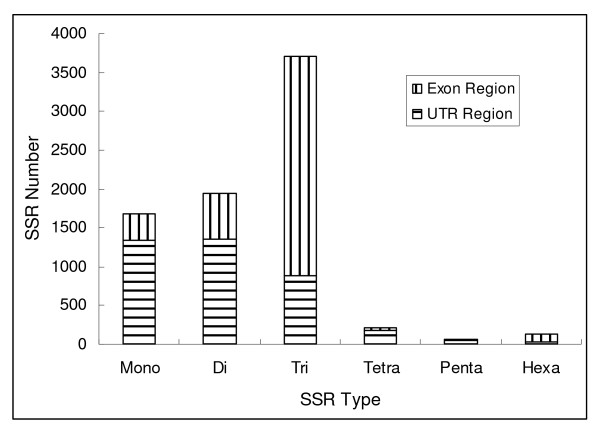
**Number of mono-, di-, tri-, tetra-, penta- and hexa- SSRs and their distribution between UTR and exon regions**.

A total of 18,928 non-redundant castor bean EST sequences trimmed were identified from 62,611 publicly available EST sequences by running the EST-TRIMMER and the CD-HIT programs. The search for microsatellites in 18,928 non-redundant castor bean ESTs representing approximately 13.68 Mb revealed 7,732 microsatellites in 5,376 ESTs; nearly one in 3.5 unique ESTs (28.4%) contained at least one SSR; 2,356 ESTs contained more than one SSR and 573 SSRs were found as compound SSRs. This corresponds to an average distance between SSRs of approximately 1.77 kb (i.e. one SSR per 1.77 kb) or one SSR-containing EST every 2.45 ESTs. The SSRs identified contained 1939 di-, 3698 tri-, 220 tetra-, 61 penta-, 138 hexa-, and 1676 mononucleotides (Table 1). The trinucleotides are the dominant motifs (Figure 1). Among motif repeats, 1624 A/T repeats accounting for 96.9% of total mononucleotide repeats (1676) were the dominant mono- motifs; 1350 AG/CT repeat accounting for 69.6% of total dinucleotide repeats (1939) are the dominant di- motifs. However, the trinucleotide motifs were relatively diverse with 321 AAG/CTT, the richest repeat among tri- motifs, accounting for 8.7% of total trinucleotide motifs (3698). Similarly, there were no obvious dominant motifs among the tetra-, penta- and hexanucleotide motifs.

**Table 1:** Occurrence of 7732 SSRs identified in a set of 18,928 non-redundant castor bean ESTs

SSR motifs	Number of repeats													
	**4**	**5**	**6**	**7**	**8**	**9**	**10**	**11**	**12**	**13**	**14**	**15**	** > 15**	

A/T							435	288	209	138	119	83	352	1624
C/G							9	14	11	6	4	3	5	52
AC/GT		49	27	11	8	11	3	2		1		2	4	117
AG/CT		623	200	130	81	43	58	29	56	38	17	25	49	1350
AT/TA		181	63	37	40	28	17	28	15	14	6	7	33	469
CG/GC		2		1										3
AAC/GTT	142	41	31	11	5		1						2	233
AAG/CTT	419	184	109	58	42	20	18	17	1			1		869
AAT/ATT	166	96	39	34	2	8	2	2	1			1		351
ACC/GGT	326	125	54	28	7	13		1						554
ACG/CGT	41	18	8	2	3	2								74
ACT/AGT	24	17	8	3	1									53
AGC/GCT	349	135	47	28	22	7	5	1						614
AGG/CCT	177	50	24	19	10	3	1		1					285
ATC/GAT	295	82	30	27	18	6		1	2					461
CCG/CGG	136	34	18	16										204
AAAC/GTTT	12	1												13
AAAG/CTTT	54	24	5	3	4									90
AAAT/ATTT	33	3		1										37
Other Tetra-*	56	17	6										1	80
AAAGA	10			1										11
Other Penta-*	44	5				1								50
Hexa-*	106	19	11	2										138
														
N							444	302	220	144	123	86	357	1676
NN		855	290	179	129	82	78	59	71	53	23	34	86	1939
NNN	2095	782	368	226	110	59	27	22	5	0	0	2	2	3698
NNNN	155	45	11	4	4	0	0	0	0	0	0	0	1	220
NNNNN	54	5	0	1	0	1	0	0	0	0	0	0	0	61
NNNNNN	106	19	11	2	0	0	0	0	0	0	0	0	0	138
TOTAL	2410	1706	680	412	243	142	549	383	296	197	146	122	446	7732

* The motif with less 10 SSR was not listed.

Inspection of SSR location on EST sequences showed that 1344 mono- repeats (accounting for 80.2%), 1362 di- repeats (accounting for 70.3%), 183 tetra- repeats (accounting for 83.2%), and 47 penta- repeats (accounting for 77.1%) occurred within un-translated regions (UTRs), while 2813 tri- repeats (accounting for 76.1%) and 101 hexa- repeats (accounting for 73.2%) occurred within expression regions (see Figure 1).

### Polymorphism and genera transferability of EST-SSRs markers

**Figure 2:**
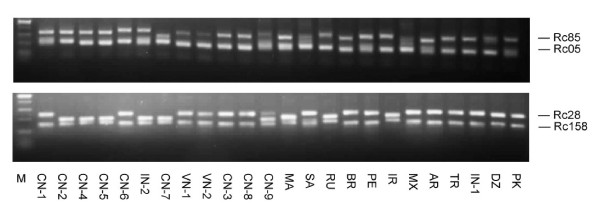
**PCR products and their length polymorphisms of four EST-SSR markers (Rc05, Rc85, Rc28 and Rc158) on agarose gel among 24 germplasms (see Table 2 for the codes of germplasms)**.

Out of 6056 SSR embedded within 3871 ESTs, excluding 1676 MNRs, primer pairs could be designed for 4223 SSR loci (69.7%) by using PRIMER3. The remaining sequences contained either too little DNA sequence flanking the SSR loci or the sequences were inappropriate for primer modeling. Three hundred and seventy-nine primer pairs flanking 151 di-nucleotide repeats (DNRs), 185 tri-nucleotide repeats (TNRs), 35 tetra-nucleotide repeats (TeNRs), 4 penta- nucleotide repeats (PNRs) and 4 Hexa-nucleotide repeats (HNRs) were assayed to test the polymorphism and genera transferability of EST-SSRs in 24 accessions worldwide (see additional file 1, Table S1, additional). In 308 (81.2%) cases, PCR products could be amplified with genomic DNA, while for 71 primer pairs PCR completely failed, amplified too weakly, or amplified multiple bands and the 71 primers were excluded from further analysis (see additional file 2 Table S2, additional). In 21 cases, the amplicons obtained were of obviously larger size than expected from the EST sequence, probably due to the presence of introns. The amplification of introns may cause problems, since fragments above 300 bp could not be scored accurately for small differences in fragment size. Additionally, it can be assumed that in several cases the observed polymorphism is caused by a size polymorphism within the intron, which may overshadow a putative polymorphism of the microsatellite. Thus the 21 primer pairs containing obvious introns and producing over 300 bp fragments were also excluded from further analyses. One Hundred and sixty-nine primer pairs were monomorphic, covering 56 di- motif loci, 104 tri- motif loci and 9 tetra- motif loci. In total, 118 polymorphic EST-SSR markers from 287 primer pairs were identified, including 68 di- motif loci, 42 tri- motif loci and 8 tetra- motif loci (see additional file 2, Table S2, additional). The proportion of polymorphic primers was 41.1%. The polymorphic proportion of di-, tri-, and tetra- motif loci were 54.8%, 28.8% and 47%, respectively. From the 118 loci we identified 350 alleles with an average of 2.97 alleles per locus (Table S3, Figure 2). Of the 350 alleles, 223 alleles were from di- loci with an average of 3.28 per locus, 107 alleles were from tri- loci with an average of 2.49 per locus. Across 118 loci, gene diversity (expected heterozygosity, *He*) ranged from 0.08 to 0.78 (mean = 0.41 ± 0.02). Among 68 dinucleotide loci and 42 trinucleotide loci, the mean of *He *were 0.44 and 0.37, respectively. Across dinucleotide and trinucleotide loci, dinucleotide SSRs were significantly more polymorphic than trinucleotide SSRs (nA and *H*e both *P *< 0.01; 2-sample *t *test). Across 118 loci, PIC values ranged from 0.07 to 0.73 (mean = 0.36 ± 0.02), suggesting the EST-SSR markers developed had moderate level of polymorphism. BLAST analyses showed that 76 EST sequences from the developed 118 polymorphic SSR markers shared significant homology to *Arabidopsis *loci. The functional annotations of markers developed were listed in Table S3 (see additional file 3, additional).

To test the genera transferability of EST-SSRs identified in castor bean to *Jatropha curcas *and *Speranskia cantonensis*, the 308 primer pairs, which could successfully amplify PCR products in castor bean were tested for amplification of the genomic DNA of *J. curcas *and *S. cantonensis *with the same PCR conditions used in castor bean. 155 of 308 (50.2%) primer pairs amplified in *S. cantonensis*, and 74 of 308 (24.0%) primer pairs amplified in *J. curcas *(see additional file 1, Table S1, additional).

### Genetic relationships among germplasms

**Figure 3:**
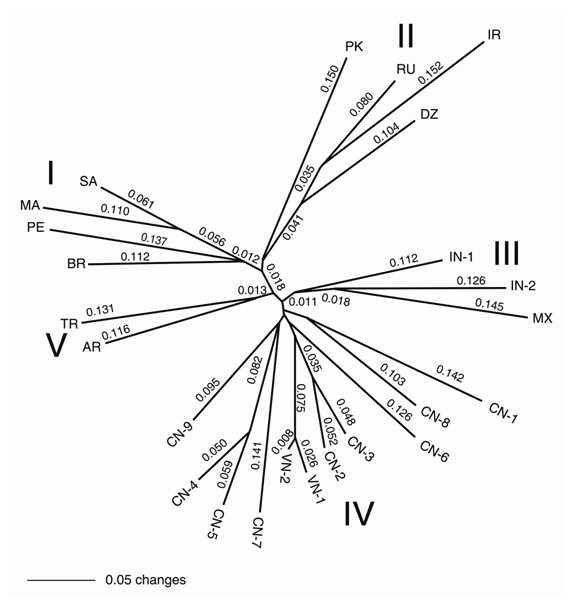
Dendrogram constructed from genetic distances estimated from genotypes of 118 EST-SSRs among 24 germplasms using the UPGMA Nei-Li criteria within PAUP*. The numbers beside lines denote the branch length (see Table 2 for the codes of germplasms).

A dendrogram based on UPGMA Nei-Li's criteria was generated with five distinct clusters (Figure 3). Cluster I included two African (SA and MA) and two South American (BR and PE) accessions; Cluster II contained one African (DZ), one Russian (RU), and two west Asian (PK and IR) accessions; Cluster III comprised of one North American (MX) and two Indian (IN-1 and IN-2) accessions; Cluster IV covered all Chinese (CN1-9) and Vietnam (VN1-2) accessions. The dendrogram based on Neighbor-Joining criteria was very similar to the UPGMA tree, and the five distinct clusters (Cluster I, Cluster II, Cluster III, Cluster IV and Cluster V in Figure 3) were again identified, though there were slight differences in branch length within clusters (data not shown).

## Discussion

### SSR frequency and distribution

The non-redundant EST sequences provided a more accurate representation of the densities of SSR motifs in the transcribed portions of the genome [20,32]. Based on the 18,928 non-redundant castor EST sequences, 7732 SSRs were identified. The overall density of SSRs is one SSR per 1.77 kb, nearly one in 3.5 unique ESTs (23.6%). Using the same cut-off criteria, Ellis and Burke inspected the frequency of EST-SSRs from 33 plant genera and found that the frequency varied from one in every 5 unique ESTs (21%) to one in every 40 unique ESTs (2.5%), with a mean frequency of nearly one SSR-ESTs in every 10 unique ESTs (9.0%) [18]. Compared to the 33 plant genera, castor exhibits considerably high frequency of EST-SSRs. To further compare the overall densities of SSRs in castor bean EST sequences with that reported in other plants, we used the same cut-off criteria as Cardle et al. [21] with 7, 5, 4 and 4 repeats for di-, tri-, tetra- and penta-, respectively, excluding the mono-repeats. Correspondingly, we identified 2710 SSRs with one SSR per 5.0 kb (1/5.0kb) EST sequence in castor. This density is higher than that in soybean (1/7.4 kb), maize (1/8.1 kb), tomato (1/11.1 kb), Arabidopsis (1/13.83 kb), poplar (1/14.0 kb), and cotton (1/20.0 kb). However, it is lower than that in rice (1/3.4 kb). Similarly, we separately used the same cut-off criteria as Aggarwal et al. used in coffee [33], Low et al. used in oil palm [34] and Feng et al. used in rubber tree [35], and identified 10,442 (1/1.31 kb), 4,177 (1/3.3 kb) and 3,616 SSRs (1/3.8 kb) respectively, higher than that in coffee (1/2.16 kb) and oil palm (1/7.7 kb), and lower than that in rubber tree (1/3.39 kb). Varshney et al. assumed that the high frequency of SSR in rice EST sequences may be due to its small genome size [36]. The genome size of castor was estimated to be 323 Mb [37]. The high frequency SSR in castor EST sequences may be related to its small genome size.

Like other plants, A/T is the main mononucleotide motif in castor bean EST sequence [23]. Among the dinucleotide repeat motifs identified, AG/CT repeats (1350) were the most common in the dataset, accounting for 69.6% of the total dinucleotide motifs (1939). These results are consistent with the frequency of DNRs identified in Arabidopsis, rice, soybean, maize, oil palm, coffee, barley, wheat and rubber tree [23,24,27,32,34,35]. Kantety et al. suggested that the high level of occurrence of GA/CT motifs could be due to the high level of occurrence of the translated amino acid products of the motifs [38]. The GA/CT motifs are translated into GAG (Glu), AGA (Arg), CUC (Leu) and UCU (Ser). We inspected the codon usage from 200 ORFs containing 44,298 codons in castor bean EST sequences and detected 10,892 codons for these four amino acids (24.6% of the total codons analyzed), accounting for that the four amino acids have a relatively higher frequency than the amino acids produced by the other dinucleotide repeats (data unshown). Thus, Kantety et al.'s assumption was supported in our study. The CG/GC is the most rare di- repeat in accordance with that reported in other plants compared [23,24,27,32,34,35].

Varshney et al. reported that among cereal species TNRs were the most frequent (54-78%) followed by DNRs (17.1-40.4%) and TTNRs (3-6%), excluding MNRs [36]. Our results (excluding MNRs) are consistent with cereal species with the most frequent TNRs (61.1%), followed by DNRs (32.0%), and TTNRs (3.6%). The abundance of trimetric SSRs in ESTs was attributed to the absence of frameshift mutations in coding regions when there is length variation in these SSRs [39]. Among the tri- motifs AAG/CTT is the most frequently occurring (23.5%) in castor bean ESTs, followed by AGC/GCT (16.6%), ACC/GGT (15.0%), ATC/GAT (12.5%), AAT/ATT (9.5%). Morgante et al.'s observation that AAG/CTT is predominant and CCG/CGG is relatively rare tri- repeats in dicotyledonous plants [23] was confirmed.

The mono-, di-, tetra- and penta- repeat loci mainly occurred within UTR regions, while tri- and hexa- repeat loci occurred mainly within exon regions. This seems to be a common feature of EST-SSRs and has often been found in other organisms. This could be a result of selection and evolution, since tri- and hexa- SSRs do not change the coding frame in coding regions when there is a SSR length variation, while mono-, di-, tetra- and penta- SSR easily change the coding frame within coding regions and give rise to negative mutation when the SSR length variation occurred.

### Polymorphism of EST-SSR markers and genera transferability

Hitherto, little work has been done on the development and application of SSR markers in castor bean genetic and breeding studies. We obtained 118 polymorphic EST-SSR markers from 379 primer pairs within 24 germplasm sampled with a polymorphic ratio of 41.1%, excluding the null allele primers and those that harbor obvious introns. Compared to other plants, the polymorphic ratio of EST-SSR primers in castor bean is at the medium level [20]. These polymorphic EST-SSR markers derived herein, to our knowledge, are the first report on development of genic microsatellite markers in castor bean to date. Using these 118 polymorphic EST-SSR markers, 350 alleles were identified from 24 accessions with an average of 2.97 alleles per marker. Allan et al. reported nine genomic SSR markers with an average of 0.403 gene diversity (PIC) and an average of 3.01 alleles per locus [13]. Bajay et al. developed 12 genomic SSR markers with an average of 0.416 gene diversity (*He*) and an average of 3.3 alleles per locus [40]. Our results displayed that the gene diversity (*He*) and PIC value of the 118 polymorphic markers were 0.41 and 0.36, respectively. These results were consistent with each another, suggesting that SSR locus of castor bean represents a moderate level of gene diversity. The gene diversity values (*He *and PIC) reported herein can serve as a guide in selecting the loci that are most likely to be informative in further castor bean research.

As mentioned above, di- and tetra- SSRs mainly occurred within UTR regions, while tri- SSRs mainly occurred within exon regions. Unsurprisingly, di- (54.8%) and tetra- (47%) motif loci presented higher polymorphic proportions than tri- motif loci (28.8%) in castor bean, suggesting that the SSRs which occurred within UTR are more polymorphic than those in exon regions. Across di- and tri- motif loci, di- motif markers presented significantly higher gene diversity than those of the tri- motif markers. These observations showed that the SSR loci harbored within UTR regions were more polymorphic than these harbored within exon regions in castor bean.

Transferability of EST-SSRs among closely related genera has been reported in many crops. Ellis and Burke summarized the transferability of EST-SSRs among plant taxa and exhibited a variation range of EST-SSRs cross-genera transferability from 10% to 80% [18]. Our results indicated that castor bean EST-SSRs had a moderate transfer rate (50.2%) in *S. cantonensis *and a relatively lower transfer rate (24.0%) in *J. curcas*. Raji et al. reported the transfer rate of EST-SSR markers developed from *Manihot *to castor bean was 15% [41]. The different cross-genera transferability of EST-SSRs may be related to the evolutionary distance between the three genera, since castor bean phylogenetically has a more distant relationship with *Jatropha *than *Speranskia *and *Manihot *[42].

### Evaluation of genetic relationships among germplasms

As mentioned above, castor bean belongs to a monotypic genus with great phenotypic diversity and phenotypic plasticity. Castor bean is a fast-growing and easily-establishing perennial shrub under various habitats, and is widespread throughout tropical and subtropical regions and is often found on wastelands today. It is difficult to establish castor bean's origin now, though it is thought to be native to the southeastern Mediterranean Basin, Eastern Africa, and India. According to Moshkin, there are four main centers of genetic variability viz., Irano-Afghanistan-USSR region, Palestine-SW Asia, India-China and the Arabian Peninsula, each with its own specific plant characteristics [43]. It is an acceptable view that castor bean landraces collected from South or North America today were most likely introduced from Africa or west Asia in early society due to human activities.

Our current research identified five distinct groups Clusters I-V within 24 samples using the genotypes of 350 alleles. Apparently, the five clusters lacked a geographic structure because the two South American germplasms (BR and PE) clustered with two African members (SA and MA) in Cluster I, and the North American accession (MX) clustered with two Indian (IN-1 and IN-2) members in Cluster III. However, if we assume that the two South American germplasms (BR and PE) and the one North American germplasm (MX) were introduced from Africa or west Asia, our current research seems to support, in a way, Moshkin's view [43], namely, Cluster I represents African members, Clusters II and III represent Irano-Afghanistan-USSR and Palestine-SW Asia members, and Clusters IV and V represent India-China members. It is noteworthy that the germplasms sampled in the current study is limited and incomplete. It remains to be determined whether this geographic pattern of germplasm group is present in a more extensive survey of germplasm samples. Allan et al.'s studies [13] did not identify distinct geographic groups among worldwide germplasms. The possible reasons could be that 1) the polymorphic markers used in their studies were limited, or 2) many castor bean germplasms were introduced or multi-introduced across several continents due to human activities. It may be difficult to figure out the origin and domestication of castor bean without the genotype of the wild castor bean germplasms. Without a doubt, the polymorphic EST-SSR markers developed herein will provide robust genetic markers for further investigation of the origin and evolution of castor bean, though the geographic structuring of castor bean germplasms detected from our current study is uncertain.

## Conclusion

In summary, the castor bean EST database harbored highly rich SSR sites and the EST-SSR markers reported herein exhibited moderate levels of gene diversity. These EST-SSR markers should prove useful for both genetic mapping and population structure analysis, facilitating breeding and crop improvement of castor bean.

## Methods

### Plant material and EST retrieval

Twenty-four worldwide accessions representing the main germplasms of castor bean from 14 countries were used to screen the polymorphism of SSR markers developed, and to investigate the genetic diversity of germplasms based on the polymorphic SSR markers. Seeds of each accession were obtained from the USDA National Plant Germplasm System http://www.ars-grin.gov/npgs/ and our collected landraces in China and Vietnam (Table 2). Phylogenetically, the genus *Speranskia *has a closer relationship with *Ricinus *than the genus *Jatropha *[42]. The genomic DNAs of *Jatropha curcas *and *Speranskia cantonensis *were used to test the cross-genera transferability of EST-SSR markers which can amplify PCR products using castor bean genomic DNA. The seeds of accessions were germinated at a greenhouse, and the young leaves were collected for genomic DNA extraction using a CTAB methodology [44].

**Table 2:** Germplasm accessions used for testing polymorphism of EST-SSR markers and inspecting genetic relationships

Code	Genbank ID	Homology in *Arabidopsis*
PI 253621	Morocco (MA)	From USDA-ARS*
PI 257461	South Africa (SA)	From USDA-ARS
PI 257654	Russia (RU)	From USDA-ARS
PI 241369	Brazil (BR)	From USDA-ARS
PI 215775	Peru (PE)	From USDA-ARS
PI 250938	Iran (IR)	From USDA-ARS
PI 255238	Mexico (MX)	From USDA-ARS
PI 277025	Argentina (AR)	From USDA-ARS
PI 167288	Turkey (TR)	From USDA-ARS
PI 248961	India (IN-1)	From USDA-ARS
PI 258388	Algeria (DZ)	From USDA-ARS
PI 250622	Pakistan (PK)	From USDA-ARS
CYB03_1-6	Yunnan, China (CN-1)	From XTBG Seed Bank
CYN01_2-1	Yunnan, China (CN-2)	From XTBG Seed Bank
CYN20_2-20	Yunnan, China (CN-3)	From XTBG Seed Bank
CYN21_2-21	Yunnan, China (CN-4)	From XTBG Seed Bank
CYN24_2-24	Yunnan, China (CN-5)	From XTBG Seed Bank
CYB04_4-1	Yunnan, China (CN-6)	From XTBG Seed Bank
INB01_5-6	India (IN-2)	From XTBG Seed Bank
CYB05_6-9	Yunnan, China (CN-7)	From XTBG Seed Bank
CYSH1_15-1	Shanxi, China (CN-8)	From XTBG Seed Bank
CYD3_15-3	Yunnan, China (CN-9)	From XTBG Seed Bank
VNBY1	Vietnam (VN-1)	From XTBG Seed Bank
VNBH2	Vietnam (VN-2)	From XTBG Seed Bank

*USDA-ARS: Plant Genetic Resources Conservation Unit (at Griffin, GA, USA); XTBG: Xishuangbanna Tropical Botanical Gardens (at Menglun, Yunnan, China)

Castor bean EST sequences were obtained via the ENTREZ search tool of the EST database at the NCBI http://www.ncbi.nlm.nih.gov/nucest. A total of 62,611 castor bean ESTs originated from different tissues were available for this study on January 1, 2009, including the 750 ESTs (GE632454-GE637384) from developing seeds [45], 158 ESTs (AM267320-AM267478) from phloem [46], 4307 ESTs (EV519634-EV523941) from endosperm [47], 4,902 ESTs (AM267321- AM267479) from developing seeds [Kroon et al. released in 2008, unpublished], 329 ESTs (CF981112-CF981441) from seed [Cahoon et al. released in 2003, unpublished], and the 11,633; 24,567; 5,619 and 10,346 ESTs (EG690439-EG702071, EG665872-EG690438, EE254600- EE260857, EG656356-EG665871, EE253769-EE254599) from developing seeds, root, flower and leaf, respectively [Melake et al. released in 2006, unpublished]. The FASTA-formatted files of EST sequences were downloaded for further data mining.

### Data mining for SSRs

In a preliminary step, polyA and polyT stretches which correspond to polyA-tails in eukaryotic mRNA were removed with the help of the EST-trimmer software http://www.pgrc.ipk-gathersleben.de/misa/download/est-trimmer.pl until no stretch of (T)5 or (A)5 was present in a range of 50 bp on the 5'- or 3'-end, respectively. EST sequences of less than 100 bp were discarded and sequences larger than 800 bp were clipped at their 3' side to preclude the inclusion of low quality sequences [27]. To remove redundant ESTs, the CD-HIT program [48] was used with a 95% sequence similarity threshold. Then trimmed non-redundant EST sequences were scanned using the MISA (MIcroSAtellite) tool [27] to identify all SSRs within a set of sequences. We set the script to identify all possible mono-, di-, tri-, tetra-, penta- and hexanucleotide repeats (MNRs, DNRs, TNRs, TeNRs, PNRs and HNRs) with a minimum of 10, 5, 4, 4, 4, and 4 subunits, respectively. The results of the MISA run were transferred to a Microsoft Excel worksheet for further analyses.

To localize the distribution of SSRs on EST sequences, the ESTscan2 http://www.ch.embnet.org/software/ESTScan2.html was used to inspect the ratio of SSR distribution on the transcribed regions (TRs) and UTRs.

### PCR conditions and separation of microsatellites

Primer pairs were designed from the flanking sequences, using PRIMER3 software [49] in batch mode via the *p3_in.pl *and *p3_out.pl *Perl5 scripts within the MISA package [27]. To test the polymorphisms of EST-SSRs identified in castor bean, we randomly selected 379 primer pairs. The target amplicon size was set as 100-300 bp, the optimal annealing temperature as 60°C, and the optimal primer length as 20 bp.

PCR primers were developed and an M13 forward (GGAAACAGCTATGACCAT) was added to the 5' end of one of each primer pair using OliGO 6.67 (Molecular Biology Insights) to determine which tag would produce the least offensive secondary structures. Inclusion of the 5'-tag allows use of a 3^rd ^primer in the PCR (M13F) that is fluorescently labeled for detection on ABI3730 DNA Analyzer. M13F primers were labeled with a FAM fluorescent dye. PCR reactions were carried out in a 10 μl volumes containing 1x PCR buffer (10 mM Tris-HCl pH 8.4, 50 mM KCl, and 2 mM MgCl_2_), 100 μM each dNTP, 0.04 μM tag labeled Forward primer, 0.16 μM universal dye labeled primer, and 0.2 μM Reverse primer, and 2 U of *Taq *DNA polymerase. Approximately 10 ng of genomic DNA was used in each reaction. The reagents for PCR amplification were from TAKARA Biotechnology (DaLian) CO. LTD.

Primers were tested using TOUCHDOWN thermal cycling programs encompassing a 10° span of annealing temperatures ranging between 65-55°C, or 60-50°C. Cycling parameters were: an initial denaturing step of 3 min at 95°C, followed by ten cycles of 30 s at 94°C, 30 s at the highest annealing temperature (annealing temperature was reduced by 1°C per cycle), 45 s at 72°C, followed by 30 cycles of 30 s at 94°C, 30 s at 55°C (for 65-55°C touchdown range) or 50°C (for 60-50°C touchdown range), 45 s at 72°C, and a final extension time of 10 min at 72°C. PCR products were initially scored for amplification on agarose gels, and successful PCR products were subsequently sized on an ABI 3730 DNA Analyzer, after clean-up with Millipore^® ^96 well filter plate. Genescan 500 ROX size standards (Applied Biosystems, Foster City, California) were run in each lane to allow for the accurate determination of fragment size, and alleles were called using the GeneMapper software V4.0 (Applied Biosystems). Ambiguous samples were run a second time.

The putative functions of identified polymorphic markers were annotated by BLASTX against the NCBI Non-Redundant Protein http://www.ncbi.nlm.nih.gov/RefSeq/. In order to test the cross-genera transferability of SSR markers developed from castor bean EST sequence, all primer pairs producing successful PCR bands using castor bean genomic DNA were tested using *J. curcas *and *S. cantonensis *genomic DNA as templates.

### Statistical analysis

The level of polymorphism per locus (number of alleles, nA, and expected heterozygosity [i.e., gene diversity], *H*e) was calculated using the program GDA [50]. The polymorphic information content (PIC) is a tool to measure the informativeness of a given DNA marker. Thus we calculated the PIC value for each locus using PIC calculator http://www.liv.ac.uk/~kempsj/pic.html. In order to investigate the genetic relationships among germplasms using these polymorphic SSR markers identified, we scored these SSR products as the presence (1) and absence (0) of the band, thus generating a binary matrix. The binary data matrix was transferred to the software PAUP to construct the dendrogram among germplasms. The unrooted dengrograms were generated with Neighbor-Joining and UPGMA Nei-Li's criteria within PAUP*version 4.0 [51].

## Authors' contributions

LQ and CY developed and screened the DNA markers, performed molecular and statistical genetic analyses, BT performed data mining analyses and assisted with developing the DNA markers, JBY assisted with molecular and statistical genetic analyses. AL designed and coordinated the study and assisted with statistical genetic analyses and drafting the manuscript. All authors read and approved the final manuscript.

## Supplementary Material

Additional file 1 **Table S1**: A summary for the primer sequences of 379 EST-SSR markers tested and their PCR amplification using genomic DNA as templates among castor bean, *Jatropha curcas *and *Speranskia cantonensis*.doc Click here for file

Additional file 2 **Table S2**: Validation and characterization of polymorphic SSR markers derived EST sequences.doc Click here for file

Additional file 3 **Table S3**: Homology with *Aradidopsis *and functional annotations of the EST-SSR markers.doc Click here for file
