# Supplementary material for: Exploiting EST databases for the development and characterization of EST-SSR markers in castor bean (Ricinus communis L.)
Source: BMC Plant Biol. 2010 Dec 16;10:278. doi: 10.1186/1471-2229-10-278 (PMC3017068; doi:10.1186/1471-2229-10-278)
Supplement: Additional file 1 — Table S1: A summary for the primer sequences of 379 EST-SSR markers tested and their PCR amplification using genomic DNA as templates among castor bean, Jatropha curcas and Speranskia cantonensis.doc [file 1471-2229-10-278-S1.DOC]

**Table S1: A summary for the primer sequences of 379 EST-SSR markers tested and their PCR amplification using genomic DNA as templates among castor bean, *Jatropha* *curcas* and *Speranskia cantonensis***

| **Code** | **ID** | **SSR**  **motif** | **Primer** | **PCR**  **amplification** | **Transferability** | |
| --- | --- | --- | --- | --- | --- | --- |
|  |  |  |  |  | ***Jatropha curcas*** | ***Speranskia cantonensis*** |
| Rc01 | EG665073 | (ga)_8_ | F: AAATAGTAGGCGAGGGTTTTCC  R: AGGGCATGAGTTATCACCAGAT | ＋(N)^a^ | －^d^ | 🗸^e^ |
| Rc02 | EG662973 | (gt)_9_ | F: CCTCCATTGGTTTCTCCATTTA  R: TGATTCGAGATCAGAGTTGACG | ＋^b^ | － | － |
| Rc03 | EG686206 | (at)_8_ | F:TGATGCTAATGGCCTACTCAGA  R: TAATAGCACCACCATCAACTGC | ＋ | － | － |
| Rc04 | EG682908 | (ct)_8_ | F:TGAAAGCCACACAATAGTCCTG  R: TGAGATTCATCTTGTGGAGGTG | ＋ | － | － |
| Rc05 | EE255832 | (ac)_8_ | F:CATCACCTCTATCCATCCGTTT  R: CCTTCATCATTGAACTCCCTTC | ＋ | － | 🗸 |
| Rc06 | EG692399 | (ct)_9_ | F:GTCCAGGTTCACCATCTTGATT  R: ACCACCGTAGAAAGTAGCATGG | ＋(N) | － | 🗸 |
| Rc07 | EG663122 | (ga)_9_ | F:GGAAGAAGAGAAACAGCAAGGA  R: GTGGAGAGCCTCGACTAAGAAG | ＋ | － | 🗸 |
| Rc08 | EG689468 | (ta)_9_ | F:CAACTTGTCTTTCTCTCCTCCA  R: CACGACCCAACAATGTATCATC | ＋(N) | － | 🗸 |
| Rc09 | EG686478 | (ct)_9_ | F:GTCCAGGTTCACCATCTTGATT  R: ACCACCGTAGAAAGTAGCATGG | ＋ | － | － |
| Rc10 | EG678906 | (ca)_9_ | F:CAGAAGAGCTCATGTGTTGTGG  R: AGTCTTGTGATCCTCACACTGC | ＋ | － | － |
| Rc11 | GE634375 | (ta)_10_ | F:TAGAAAGAAAGCGACCCTTCTG  R: AAGAAGGAGGAGGAGGAGAAA | ＋ | 🗸 | 🗸 |
| Rc12 | T14845 | (ac)_11_ | F:ACATTATAGCGAGTGTGCTTGC  R: TGGACTTCTCAAAGCTGCATTA | ＋ | － | － |
| Rc13 | EG693832 | (ga)_12_ | F:CTGGCATTTTCTCTTTCTGGAT  R: CCGTTGTATTTGCCATTCTTCT | ＋(N) | － | － |
| Rc14 | EG666688 | (ta)_13_ | F:TTCTGATGACATTCCCAGAAAC  R: AGATATCTGGGGAGACGAAGC | ＋ | － | 🗸 |
| Rc15 | EG693665 | (ag)_14_ | F:GGTTTTAGTTGTGTGTGCAGGA  R: TCGTTTTGCATAACTCCGACTA | ＋(I)^c^ | － | － |
| Rc16 | EE259292 | (tc)_15_ | F:CATGAACCCCACAATTTACAGA  R: GACAGACCCATCTCAAGATTCC | ＋ | － | 🗸 |
| Rc17 | EG674726 | (at)_16_ | F:CCTCCCAAATCATAGTCCATTG  R: GATCAAGGTGATTAGGGACCAA | ＋ | － | － |
| Rc18 | EE260818 | (ct)_17_ | F:AAGCAATGGAGGTGAGAGAAAG  R: AGCAAGCAGTCAACCAGTGATA | ＋(I) | － | 🗸 |
| Rc19 | EG670576 | (at)_18_ | F:TCGTCCTCCCTGCTTTTATTTA  R: GCACCAAATCTTTTTAGGCAAG | ＋ | － | 🗸 |
| Rc20 | EE259262 | (ag)_20_ | F:CGTTTAAGGATCCGCATATCTC  R: GCTCAACCACCTCAAGATTTTC | ＋ | － | 🗸 |
| Rc21 | EE256518 | (at)_20_ | F:GCTTTCAGTTTCCATGTGATGA  R: GATGATCACGTCACTCTTCTGC | ＋(N) | － | － |
| Rc22 | EE256592 | (at)_22_ | F:ATGAAGTTCCAGCGTAACCATT  R: ATCTGAAACCATGGGTCATCTC | ＋ | － | － |
| Rc23 | EG662293 | (tc)_22_ | F:GCCGTCCTATTTCTCTCCTTCT  R: TTTCTACATCGGCAGTGAAAGA | ＋ | － | － |
| Rc24 | EE255894 | (at)_23_ | F:GAGAGCGTGGCTTCTCTACTTC  R: CAGTTTCACACCAAGCTGGTAA | ＋ | 🗸 | 🗸 |
| Rc25 | GE633816 | (ct)_23_ | F:ACCCCTGCAAAACCCTAATAAT  R: TTCGGTGTAAAGAATCCGACTT | ＋ | 🗸 | 🗸 |
| Rc26 | EG664176 | (at)_27_ | F:CACTTCTTCCTGATGCTCTCCT  R: AGGGGTGAAGGGATTTGTTTAT | ＋ | － | 🗸 |
| Rc27 | EG684662 | (at)_28_ | F:AGATCTTGGAGGCTGTTGTCAT  R: CTGCTTCAGGATCTCGAAAAGT | ＋ | 🗸 | 🗸 |
| Rc28 | EE260610 | (at)_29_ | F:CCCAAGTCCCAGATCTCTCTAA  R: CAGAAGAAGCAGTAGCAGCAA | ＋ | － | 🗸 |
| Rc29 | EE257519 | (at)_32_ | F:ACGAGGCTCAGACTTGTGTCTA  R: GCTGCTAATTGGCTGAAATTG | ＋ | － | － |
| Rc30 | EG674966 | (ca)_41_ | F:CGCTCTGGTGATATACGAACAC  R: TGATCGACCAAGTGAAGATGTC | －^c^ | － | － |
| Rc31 | GE632365 | (ta)_11_ | F:GCAGCTAGCCTTATTGAGGAAA  R: GAAAATATCTCCACCACCTTGC | ＋ | － | － |
| Rc32 | EE254685 | (ta)_23_ | F:CAGTTTCACACCAAGCTGGTAA  R: GTTAAGGCAGGTAACGATACGG | ＋ | － | － |
| Rc33 | GE632805 | (tc)_10_ | F:CTTGGCAGGTCACAAAGAACTA  R: GCCGTCCAGAATTGATAGGTAG | ＋ | － | － |
| Rc34 | EG691859 | (ct)_12_ | F:TACCACCAACCGCCTTTAGTTA  R: TAAGAACACAGGTCTGCTGCAT | － | － | － |
| Rc35 | EE260187 | (ag)_13_ | F:AAGCGAATTCCATACTCCTTCA  R: AAACCCACGTCTATCCTCAAAA | ＋ | － | － |
| Rc36 | EG684267 | (ta)_14_ | F:CTCCCTCTCCTCTTCATCTTCA  R: AGAACCAACCCAAAACACAAAC | － | － | － |
| Rc37 | EG695934 | (ag)_16_ | F:ATAGCTTTGCTGATTCGAGGTC  R: TCTCTCGAAACGGATAACACCT | ＋(N) | － | － |
| Rc38 | EG668661 | (ag)_17_ | F:TTTCGACCTCTACTGTTTTCCA  R: GGCGAGTCTAAATCCGAGTTATT | ＋(N) | － | － |
| Rc39 | EE260260 | (ct)_18_ | F:ATACACTCAAGTGCCCTCCATT  R: GTAACAACCCCCAGATCTGAAA | ＋(N) | － | － |
| Rc40 | EE254189 | (ag)_15_ | F:GCATCATCACAGCCTCAAATTA  R: TATCTGAGCCTCCTCTTTCCAG | ＋ | － | － |
| Rc41 | EE259194 | (gaa)_5_ | F:CTACTCCACTAAACCCGTTTGC  R: AGAGCCCTTTTGCTACAATCAC | ＋(N) | 🗸 | 🗸 |
| Rc42 | GE634109 | (aac)_6_ | F:CATTTCACCAGTTCACACACCT  R: TTTACAATCAGGGCAATACGTG | ＋(N) | － | － |
| Rc43 | EG695454 | (aga)_6_ | F:CCCACCTTTCTGAATCATCCTA  R: CGATCTCCTCAAGTGCTTTTCT | ＋(N) | － | － |
| Rc44 | GE632495 | (ata)_6_ | F:TATCAACTCCTCAGCCATTCAA  R: TCTGATGAGCTCTTCTCTGCTG | － | － | － |
| Rc45 | EG698508 | (cac)_6_ | F:TATTACAATACCGCCCCTATGC  R: ACCGCTGTTATTCCTGTCAAAT | ＋ | － | － |
| Rc46 | GE634091 | (cag)_6_ | F:GAAGCAACTGGCAATGAAGC  R: GATTGTCGAATTTCTCCCACAG | － | － | － |
| Rc47 | EG694792 | (ctg)_6_ | F:CACCCAGCTAGATCATTCAACA  R: AGCAGCAGATTTTGAGAGAAGG | ＋(N) | 🗸 | 🗸 |
| Rc48 | EG695946 | (ctt)^6^ | F:TCGAAACTCTCTTCTTCGCTTT  R: GCAAAGGTATCCTTGAAACCAC | ＋(N) | － | － |
| Rc49 | GE632944 | (gaa)_6_ | F:CACCATCTCACCTACTTGTCCA  R: CACTAGGTCCAACGCTTTCTCT | ＋(N) | － | 🗸 |
| Rc50 | EG681212 | (gac)_6_ | F:GTTTTGCACTCTTCTGGAGGAC  R: TTCTGGCTCAAAATCACTAGCA | － | － | － |
| Rc51 | EG664367 | (gcc)_6_ | F:AGGACTTATTCGACCCAGATGA  R: GATGAGTACTGAAGCGATGGTG | ＋ | － | － |
| Rc52 | EG680302 | (gga)_6_ | F:GTGTGCAAAGAGAGAGGAGGTT  R: CTACCTTGGTTGTTCTCGCAAT | － | － | － |
| Rc53 | EG697581 | (tgc)_6_ | F:TGCAAGAACAAGATTGAGTCCA  R: GGCTCCAACAATAACAAAGGAG | ＋ | － | － |
| Rc54 | EG682008 | (tta)_6_ | F:CTGTGTTCTTGCCTCCTCTCTT  R: TTTCTTTCTGCTACTGGGTGTG | ＋(N) | － | － |
| Rc55 | EG682520 | (ttg)_6_ | F:TTAGCAACAACTCTTTGCGTGT  R: TCCATCTAATGATCCCCATCTC | ＋(N) | － | － |
| Rc56 | EG694392 | (aga)_7_ | F:TCCTTCTGCCAAAAGACCTAAC  R: AAAGGTGATGAGAGTTCCGAGA | ＋(N) | － | － |
| Rc57 | EG689318 | (atc)_7_ | F:TGTAGCAGAGAAATGCAAGGAA  R: ATTTAAGGTCAGCCCAAGAACA | ＋ | 🗸 | 🗸 |
| Rc58 | GE632335 | (cag)_7_ | F:CAGAACCCTAACCCTAACCCTAA  R: CAAGCATCCTGATAAGCCATCT | ＋(N) | － | ＋ |
| Rc59 | GE635877 | (ctg)_7_ | F:GTTAAGAAAGACGATGGCAACC  R: ACATGGCTGGTCTGGTTCAT | － | － | － |
| Rc60 | GE636524 | (gaa)_7_ | F:GTTGCCTTTCAGATCTTCCATC  R: TGTGCTGATAGACGACTTTTGG | ＋(N) | － | － |
| Rc61 | EG662586 | (ggc)_7_ | F:GCACTGAGGGTTAATTCTGGAC  R: GTCGTAGACCACCTTAGCATCC | ＋ | 🗸 | 🗸 |
| Rc62 | EG694958 | (tcc)_7_ | F:GTTACTTCTAACGGCCAGATCC  R: ACAAACAATAACAGCACCATCG | ＋(N) | － | 🗸 |
| Rc63 | GE637048 | (tga)_7_ | F:CTAATATTGCCAAACCCGAGTC  R: TGTCTCCTCATCCTCACTCTCA | ＋(I)^c^ | － | － |
| Rc64 | GE633090 | (aag)_8_ | F:TTCTTAAAGGCGAATTCTCTCG  R: TTCTCATCAGCAGCATCATCTT | ＋(N) | － | － |
| Rc65 | EG701975 | (aga)_8_ | F:CGTAGCTGAAGCTGAACAAAGA  R: GCTTGACGGAGAGTAGAGCAAT | ＋ | 🗸 | 🗸 |
| Rc66 | EG697360 | (gag)_8_ | F:ACAGACCCCATAGAGGAACTCA  R: GCTGCACAGTTCATTACGACTC | ＋(N) | － | － |
| Rc67 | EG700671 | (tca)_8_ | F:ACAGCATTTCCAGCTATGTCAA  R: CCAGCCTCGGTATCAATTAAAG | ＋(N) | － | － |
| Rc68 | EG695217 | (tct)_8_ | F:AAGAAGAAGGAATCCCACAGC  R: CAGCAACACCGACACTACAGAT | ＋(N) | － | 🗸 |
| Rc69 | EG697256 | (ttc)_8_ | F:TGGCAAGAACCAACAAGTACAC  R:AGGTGAAAGAGGAGGTGTGGTA | ＋(N) | － | 🗸 |
| Rc70 | GE636935 | (ata)_9_ | F:TGTAGCACCAACACCAAGTACC  R: TTGCAGAGCGTAGATGCTAAAA | － | － | － |
| Rc71 | GE636753 | (gaa)_9_ | F:CGACCTACCAGCCTACCTGAC  R: GGTAGCAGCGGAAGAAAGTC | － | － | － |
| Rc72 | GE636357 | (agg)_9_ | F:GCCATTTACTGCATTGTTGCT  R: GCATATCCTTAAACCCACCAAA | ＋(N) | － | － |
| Rc73 | GE633761 | (cca)_9_ | F:AGCCTGAATCCTCTGATGTAGC  R: GGTCCTGGTTCAGGATCAGTAA | ＋ | 🗸 | － |
| Rc74 | EG698249 | (ctg)_9_ | F:CTGAAGTTCCAGAGCTTCCTGT  R: TGAGAGGAAGGATTACGGAGAC | ＋ | － | － |
| Rc75 | EG697704 | (taa)_9_ | F:CAAACCCAGTCGCTAAAGAAAC  R: AGAAGCCATCTCATCTTCTTGC | ＋(N) | － | 🗸 |
| Rc76 | EG696213 | (ata)_9_ | F:GGAGGGATATGGATTTCAGATG  R: CGGAAGATGAGAGAACAGGTT | ＋ | 🗸 | － |
| Rc77 | EG662571 | (ctc)_9_ | F:CCTATCTCTGCAGCATGTGTGT  R: GGAGGTTGGTGGGTAGTTGTAG | ＋(N) | 🗸 | 🗸 |
| Rc78 | EG695459 | (cag)_10_ | F:GAAGTCTTTACTCCCACTGAGCA  R: TGAAGTAGCCCAACCACCTATT | ＋(N) | － | － |
| Rc79 | EG694574 | (aca)_10_ | F:CATCCCTTTGCAACCTAACAAT  R: GGTATTGTAGAGGGGCTTGTTG | ＋(N) | － | － |
| Rc80 | EG693130 | (ctg)_10_ | F:TGAAGTAGCCCAACCACCTATT  R: GAAGTCTTTACTCCCACTGAGCA | ＋(N) | － | － |
| Rc81 | EE254390 | (cag)_10_ | F:GAAGTCTTTACTCCCACTGAGCA  R: TGAAGTAGCCCAACCACCTATT | ＋(N) | － | － |
| Rc82 | EG659242 | (ctg)_10_ | F:TGAAGTAGCCCAACCACCTATT  R: GAAGTCTTTACTCCCACTGAGCA | ＋(N) | － | － |
| Rc83 | EG684045 | (aag)_10_ | F:CATCTCCAGATGAAAAGCCACT  R: CGCTCTCTGAGACAATGAAATG | ＋(N) | 🗸 | 🗸 |
| Rc84 | EG695475 | (aag)_11_ | F:ACCTCTCCTTCCCTCATCTCTC  R: GAATCTAACGGTGCTGATCCTC | ＋ | 🗸 | － |
| Rc85 | EG660729 | (cac)_11_ | F:GCCACTATGGTGTCATCTCGTA  R: CTTTTAATTTTAGGCGGTGGAG | ＋ | 🗸 | － |
| Rc86 | EG681190 | (cag)_11_ | F:GCAACTTCAGATGCAACAACTC  R: TCATACATCTTTGTTGCCAAGG | ＋ | － | 🗸 |
| Rc87 | EG687367 | (taa)_11_ | F:GCCACTTCCGATTATTTCTTTG  R: CGGTTCACACCCATCTCTTTAT | ＋ | － | － |
| Rc88 | EG667050 | (tct)_11_ | F:CACCAATCTCATCACCAACACT  R: GAATTGTGGGGTTTAGGGTTT | ＋ | － | 🗸 |
| Rc89 | EG679278 | (ttc)_11_ | F:TCTGTTAAACAACGGGAAAACC  R: CTACAAGAACAAAACGCCACCT | ＋ | － | － |
| Rc90 | EG666840 | (gaa)_11_ | F:CACCTCCACAAGAACAAGAACA  R: CGGACGACGAACTTTTAGATTC | ＋ | － | 🗸 |
| Rc91 | EG665468 | (gag)_12_ | F:AGTGGCATGGGCATATTATGTT  R: AACTCTCTAAGCCCCAAATTCC | ＋ | － | 🗸 |
| Rc92 | GE633562 | (gcc)_12_ | F:AGCGACACTTTGCCAAGAAG  R: AGCTTAGCCGAGTACCTCTCGT | － | － | － |
| Rc93 | EG663259 | (ttc)_12_ | F:TTTTCACTTGCACGATACCAAG  R: CGTTGAAACAACTCCTGAAATG | － | － | － |
| Rc94 | EG691818 | (gaa)_15_ | F:ACGCCACAAACACACATATCAT  R: CAAGTGCATCTGAAACGACAAT | ＋ | － | － |
| Rc95 | EG699176 | (caa)_19_ | F:TAGACCCAGCAGTGCTCATAAA  R: CCACCTTTAGGTTGGATGTTGT | ＋ | － | － |
| Rc96 | EG693205 | (aaag)_6_ | F:ATGGCAACTGTTGAGGTAATCC  R:CTACATGGCAGCACCAAGAAT | ＋ | 🗸 | 🗸 |
| Rc97 | EE258431 | (ttct)_6_ | F:TATTTCCTGCCCTTGTAGCTCT  R: AAGTTGCAAGAACACCCAAAAC | ＋(N) | － | － |
| Rc98 | EG667740 | (ttat)_7_ | F:CTTCACTTGGGCTTTTAGCTGT  R: TTATCCCCTTGTTTGGAAGTCT | － | － | － |
| Rc99 | EG686707 | (acat)_30_ | F:GGGTTCATTTTATTCCAGAACG  R: GGGATTGAAGAGGATATTGAA | － | － | － |
| Rc100 | EG681785 | (atca)_6_ | F:CCAAGAAAACAACTCCGGTTAC  R: GAGGACATGGGTTGTTGTTGAT | ＋ | － | － |
| Rc101 | EG678591 | (ag)_7_ | F:CGGATAGCGATCTCTCTTTTTC  R:ACCGTACAACACAATCGGAAAT | ＋(N) | 🗸 | 🗸 |
| Rc102 | EG672190 | (cg)_7_ | F:GCATTGATTCTATGGCTGCAT  R:TTGTTGATCGGTGAACAGTTTC | ＋(N) | － | ＋ |
| Rc103 | EG678636 | (ta)_7_ | F:CCAGAAATGCATCTGGATCG  R:TGGACTGGAGATGCTGATTATG | ＋(N) | 🗸 | 🗸 |
| Rc104 | EG686206 | (at)_8_ | F:TGATGCTAATGGCCTACTCAGA  R:TAATAGCACCACCATCAACTGC | ＋ | － | 🗸 |
| Rc105 | EG696311 | (ga)_8_ | F:GAAGGAAAGGTGTGGTTTCTTG  R:TGAAAAGAGAAAGGTGTCAGCA | ＋(N) | － | 🗸 |
| Rc106 | EG661383 | (ta)_8_ | F:GCAGGAATGTTATTCCGTTCTC  R:CTCTTAGCAACCACGTTCTCCT | ＋(N) | － | － |
| Rc107 | EE253894 | (ga)_9_ | F:GAAAAGCATCCTCTTCTGCCTA  R:ATAGAGCCCAAATTGGTGAGTG | ＋(N) | － | 🗸 |
| Rc108 | EG665105 | (ct)_9_ | F:AACGATGATCTAACAGGACACG  R:CATTGTTGTTTGGAGGAGACAA | ＋(I) | － | 🗸 |
| Rc109 | EG662014 | (ta)_9_ | F:GCAATAGTGCAGTTTGGAAGTG  R:AGAGGAGAAGAGAAGAGCGTGA | ＋(N) | － | 🗸 |
| Rc110 | EG667772 | (ac)_9_ | F:TCAGCTGAAGGTTGTGAAACTC  R:AGTGTCGAGTCGGAGGAGATAG | － | － | 🗸 |
| Rc111 | EG689201 | (ag)_10_ | F:TCCAAGAACATGTGAACCATCT  R:CGATCATGGCTTCTTTCATCTT | ＋(N) | 🗸 | 🗸 |
| Rc112 | EE254243 | (ta)_10_ | F:TATCTGAGCCTCCTCTTTCCAG  R:GGAACGACAAACACCACAACTA | ＋ |  | 🗸 |
| Rc113 | EG679802 | (ag)_11_ | F:TCAGTTGAACTCAAGGTCAGTCA  R:AAGCAGAGTGCTTCTTCAATCC | ＋ | 🗸 | 🗸 |
| Rc114 | EG675931 | (ct)_11_ | F:ATTTCTTTGACTCCAACCCAGA  R:ATTGATCAGCTTGTGGCTCAG | ＋(N) | 🗸 | 🗸 |
| Rc115 | EG686897 | (ta)_11_ | F:AGAACCGTGCAGTGGTCTTAGT  R:TGGGATTGTGATAAGTGCTTTG | ＋(N) | 🗸 | 🗸 |
| Rc116 | EG689244 | (ag)_12_ | F:TCCTTTCAGACCGAAGGAGATA  R:ATAACCATAACCCCATCCATCA | ＋ |  | － |
| Rc117 | EG691558 | (at)_12_ | F:CGTTTCCCTACGCTTTCTACAT  R:TGATTCAGGTCCCATATTAGCC | ＋ | 🗸 | － |
| Rc118 | EG689201 | (ga)_12_ | F:TCCAAGAACATGTGAACCATCT  R:CTTGCATGGTTAGTGGAGAAGA | － | － | － |
| Rc119 | EG663853 | (ag)_13_ | F:CAAACTAATCACACGCTTTCCA  R:ATTCTTCGTCGTCTTCTTTTGC | ＋ | 🗸 | 🗸 |
| Rc120 | EE255149 | (ct)_13_ | F:TATGAATGATTGGTCCCACCAG  R:CACCACAAGACAAATGGAAGAA | ＋(N) | － | 🗸 |
| Rc121 | EG697485 | (tc)_13_ | F:GCCACCATGTGAATTATCCTCT  R:TGTGAGAAGGAGAGGGTTTCAT | ＋ | 🗸 | － |
| Rc122 | EG669444 | (ct)_14_ | F:CTGTCAAAACATTCTTCGCATC  R:AATGTTGTTGGGATGAATAG | ＋ | 🗸 | － |
| Rc123 | EG689468 | (ga)_14_ | F:CAACTTGTCTTTCTCTCCTCCA  R:CACGACCCAACAATGTATCATC | ＋(N) | － | 🗸 |
| Rc124 | EE256105 | (ag)_15_ | F:GACAGACCCATCTCAAGATTCC  R:CATGAACCCCACAATTTACAGA | ＋ | － | － |
| Rc125 | EG659605 | (tc)_15_ | F:ACTCTCAACTCCAACCTTCCAA  R:ATAAAGCATCCATTGCTTCCTC | ＋(N) | － | － |
| Rc126 | GE632416 | (tg)_15_ | F:CAGTATCACGCTGTGGTTGTTT  R:CTTTGCCGACTGGAATATCAAC | ＋ | 🗸 | － |
| Rc127 | GE632416 | (ag)_16_ | F:TGCAATAATTCCCTCAGGATCT  R:CATCAAGAATACCCTCCCTGAA | － | － | － |
| Rc128 | GE635894 | (ta)_16_ | F:TTGGGGACTATGATCAAACTGA  R:TTTAAACTGCCATCAACGACAC | ＋ | － | － |
| Rc129 | EG689641 | (tc)_16_ | F:TACTGCAACTCAATCCACTGCT  R:GATAGTGCCTTTGCCTCTTTTC | ＋ | － | － |
| Rc130 | EG692156 | (ct)_17_ | F:TGAACTCACAATCAGTCCCTTC  R:TCTGGTACAGGTGATGATTTCG | ＋ | － | － |
| Rc131 | EG685152 | (ta)_17_ | F:GATCATTTGCATGGCTGAAGTA  R:CCATAGCTTTGGACACACTCAC | ＋ | － | － |
| Rc132 | EG694164 | (tc)_17_ | F:TCTTCACAGCTATTGGCAACAC  R:GGAAGCTAGGGATTAGGGATTG | ＋ | 🗸 | 🗸 |
| Rc133 | EG660924 | (ct)_18_ | F:TAGTGAGGGTCCCAGAACTGAT  R:TCAGTTCCAAAACGTCAATGTC | － | － | － |
| Rc134 | EG688615 | (ct)_18_ | F:ACAGAGAGAGAACGCACTGTCA  R:CCATCACTTCCCATTCTCCTTA | － | － | － |
| Rc135 | GE634416 | (ga)_19_ | F:CTGCTTCTCTCTGCATTGTGTA  R:CCATTCCTTCTCTGGTTTCTTG | ＋ | 🗸 | 🗸 |
| Rc136 | CF981327 | (ag)_20_ | F:CTTCTTCACTGCAAAACACCAC  R:TCTTCTCACTGTTGTTGCTGGT | － | － | － |
| Rc137 | EE260176 | (tc)_20_ | F:ATTCGGCACGAGGAGAGAACTA  R:GAGAAGGTCATGGATGGAAGAG | － | － | － |
| Rc138 | EG680179 | (tc)_21_ | F:CTTCCCAACAGCTCAATTTCTT  R:CACATGCATACATAAACCAGCA | ＋ | 🗸 | 🗸 |
| Rc139 | EE254161 | (at)_23_ | F:AGGTAACGATACGGTGTTGGAG  R:CAGTTTCACACCAAGCTGGTAA | ＋ | 🗸 | － |
| Rc140 | EE259204 | (at)_24_ | F:TCTAAGATGGTCTCGCCTCATT  R:GACCCATATAGGGCAAGGTTTA | ＋ | － | － |
| Rc141 | EG700448 | (ag)_25_ | F:AATTGCCAGCCAAACATAAGAT  R:TAAATTGGGTTAATGGGTTTGG | ＋ | 🗸 | － |
| Rc142 | GE635772 | (att)_5_ | F:ATCTAGTACGTCGGCGTTTTGT  R:AGTAACCATACGAGCACCGAAT | ＋(N) | － | 🗸 |
| Rc143 | |GE635389 | (gcc)_5_ | F:CTCTTCAATTGTGTCGCCAGT  R:TCGCTATCGTAGTCCTCTTCGT | ＋(I) | － | － |
| Rc144 | GE635813 | (gct)_5_ | F:GATAGAAGCGAAGTGTCCGTGT  R:TAAGTCCCTTTGAGGTCGAGTT | － | － | － |
| Rc145 | GE636673 | (tca)_5_ | F:ATCACTCCAACCACAACAACAG  R:AACAATTCCTGCAACTCCCTTA | ＋(N) | － | 🗸 |
| Rc146 | GE636623 | (tga)_5_ | F:CAGGTCCATCATCAACAAAAGA  R:GGCCAGGTACAAAACCAGTAAC | ＋(N) | － | 🗸 |
| Rc147 | GE637053 | (tgc)_5_ | F:AGACTTTCAAGAGCAGCGAAGT  R:AGTTGGCAGCCCAATTACTG | － | － | － |
| Rc148 | GE636227 | (aag)_6_ | F:CACCGTACGCTTTCTCAAATCT  R:GAGACTTCGGGACGAGAAGATA | ＋(N) | － | － |
| Rc149 | GE634416 | (agc)_6_ | F:ACAAGCCAATGAACAACAACAG  R:CCAACAAAAAGGATATGGAGGA | ＋(N) | 🗸 | 🗸 |
| Rc150 | GE634033 | (agg)_6_ | F:AGAAGCAGCAATGTAAGTGCAA  R:TGATGTTAGTGCTCCTGTTGCT | ＋(N) | － | 🗸 |
| Rc151 | GE634842 | (cag)_6_ | F:CACCGTACGCTTTCTCAAATCT  R:AATATCAGTGCCGATGCTATCC | ＋(N) | 🗸 | － |
| Rc152 | GE633725 | (cca)_6_ | F:CCCCCTCTCTCTCTCAATTTAT  R:GCTTGGAAAGAGAGATTTGATG | ＋(N) | － | 🗸 |
| Rc153 | GE632969 | (cct)_6_ | F:CGGTCACTCCATCAAATCATC  R:CGGTCCAGGTCCTCTCTATCTT | － | － | － |
| Rc154 | GE636252 | (ctg)_6_ | F:ATGCTGAGCTTCCCGAAATAG  R:GCTCTCAGCTGACGGAAGTCTA | － | － | － |
| Rc155 | GE635133 | (ctt)_6_ | F:AGAAGCAAGATCCTCACTCTG  R:AAGAGCGTTCTTGACATCATCA | ＋(N) | － | 🗸 |
| Rc156 | GE633342 | (gat)_6_ | F:GGCACACTTGTTGAGAGACTTG  R:CAAGTCCTTTAAGGCCCTTTTT | ＋(N) | － | 🗸 |
| Rc157 | EG686946 | (aat)_7_ | F:AAACAACCGAAAAGTCGAGAAG  R:TCTCCAAATAATGGAGCTGGTT | ＋(N) | 🗸 | 🗸 |
| Rc158 | EG693883 | (acc)_7_ | F:CCCCGACGATAACTACCATAAA  R:AGGTCATCGAATAACGACCAAG | ＋ | － | 🗸 |
| Rc159 | GE636194 | (agc)_7_ | F:GTTTCGATCTAAGCTTGCCAAC  R:CTTTCACCACAACTGACGCTAC | － | － | － |
| Rc160 | GE632700 | (agc)_7_ | F:CAGGAAATACCTGCGACTGCT  R:AGTCTTTTGTATTTGCCCGAAG | － | － | － |
| Rc161 | GE635922 | (cag)_7_ | F:CCCTGTTCTCCATACACTGGTC  R:GGGTTCATGGCTGAGTAGTACA | － | － | － |
| Rc162 | GE634548 | (ctc)_7_ | F:TCAAAATGCCTTCCAACTACAC  R:TATGTTCCAGGGCTTTGATCTT | ＋(I) | － | 🗸 |
| Rc163 | GE634768 | (gaa)_7_ | F:TCATGTCCACATCCTCTCAATC  R:ACTCGCGATGAATTACCTAACG | ＋(N) | － | 🗸 |
| Rc164 | GE635707 | (gca)_7_ | F:GTCTCCACAGCATCAGAATCAG  R:AGTGTTGCTTGTGGTATTGCTG | － | － | － |
| Rc165 | GE635881 | (gca)_7_ | F:ATTCCTTTCACACACGCTCTCT  R:GTATTTTAGGGGAATGGGGTTG | ＋ | － | － |
| Rc166 | EG684973 | (ggt)_7_ | F:AGTTGCAGTTGAGGTTGTGATG  R:AAGGCCTCAGTTAACCAATGAA | ＋ | － | － |
| Rc167 | GE632520 | (ttc)_7_ | F:GTCTGTGACAACAACCACGAAC  R:GAGATGGGAGAGATGAAGAGGA | ＋ | － | 🗸 |
| Rc168 | GE633169 | (aga)_8_ | F:TTCTTCCTCCTCCTCCACATAA  R:GAGGAGAAATGAAGCATTACGG | ＋ | － | 🗸 |
| Rc169 | GE635147 | (atg)_8_ | F:TGATGGCTTTAACAGTGGAGAA  R:CAGGCCTCACTGGATACTGATA | － | － | － |
| Rc170 | GE634768 | (cag)_8_ | F:GTCAAAATGACAGCGAGTTCAG  R:ATTTAAGCGCGTCAAGAGTACC | ＋(N) | － | 🗸 |
| Rc171 | EG670294 | (cca)_8_ | F:CCGCTCACTACAAGTGGTACTC  R:GTAATGTAAGGGGTTCGGTGAT | ＋(N) | － | 🗸 |
| Rc172 | GE633649 | (cgg)_8_ | F:CGAGAAAGAGCGCTAGAGAAAG  R:CAGAACGGATCAAAGAATCCTC | － | － | 🗸 |
| Rc173 | GE636104 | (cta)_8_ | F:AGAACGCAACTAACCGTCTCTC  R:GGGTCAGTGTAGACATGTTGGA | ＋(N) | － | 🗸 |
| Rc174 | EG695367 | (gag)_8_ | F:GGGTATCGAGGCTGAAATTCTA  R:AGCAACATTTTCACCAGTCTCA | ＋(N) | － | 🗸 |
| Rc175 | GE635072 | (tcc)_8_ | F:TTGGTGTTTTTGGAGAAGTGTG  R:CTAAAGCTCCGCTGAGAAACTC | ＋(I) | － | 🗸 |
| Rc176 | GE633829 | (tct)_8_ | F:TACCCAATGACTGCAATCTTCA  R:GGGTTTGTTGAGAAGGAGAAAA | ＋ | － | 🗸 |
| Rc177 | EG664236 | (acc)_9_ | F:TCGCTCTCACTGTCTCTTTCTC  R:GCCAGAATCCTTTTAACACCAG | ＋(N) | － | － |
| Rc178 | EG691843 | (aga)_9_ | F:CAGAAGCAGCAACATCAAGAAG  R:CTGTTCTTTTCCAAACGGTTCT | ＋(N) | － | 🗸 |
| Rc179 | GE633933 | (agc)_9_ | F:GACTTCATTTCGTCCAGTTTCA  R:ACAATATCGACGGAGATGGTCT | － | － | － |
| Rc180 | EG659942 | (ctt)_9_ | F:AAGAGAAACGAAACGCCATGT  R:CATACTTTCCACGTCATCTCCA | ＋(I) | 🗸 | 🗸 |
| Rc181 | EG694260 | (gac)_9_ | F:GAGACCACCAACCCTGAAGTAG  R:TCACTTCTGCTCTGCTTTGAAC | ＋(I) | － | 🗸 |
| Rc182 | GE635438 | (tct)_9_ | F:GCCAATCTGTTCACATACCTCA  R:GTCGAACTTTCATACCGGAAAA | ＋ | － | － |
| Rc183 | EG661977 | (ttc)_9_ | F:GTTGATGCTTTCTCCATCTCTG  R:GCAGAACCCAGTTCAATTTCAT | ＋(N) | － | 🗸 |
| Rc184 | EG671871 | (ata)_10_ | F:CGCATCTTGCATTTCTTTTGT  R:AGGATGGCCTGTGTCTAATAGC | ＋ | － | － |
| Rc185 | EG661982 | (ctt)_10_ | F:ACAAGATGCAGCTTTCTTGGAC  R:ATGGGAATCTCCATGAACAAAC | ＋(N) | － | 🗸 |
| Rc186 | EG683300 | (gga)_10_ | F:AGATCAAATCGGGGAAGAGAAT  R:CCATCATCAGTAGGTGGCATTA | ＋ | 🗸 | － |
| Rc187 | EG664396 | (tct)_10_ | F:GCAACACAACACAACACAACAA  R:TCCTGTATCTCCGATGATGATG | ＋(N) | 🗸 | 🗸 |
| Rc188 | EG662044 | (ttc)_10_ | F:TCTTCTTCGCCTCCAATAAAAC  R:TGTAGGGACAAAGCGATTCATA | ＋ | － | － |
| Rc189 | EG666688 | (ttc)_10_ | F:CCAGAAATGCATCTGGATCG  R:TGGACTGGAGATGCTGATTATG | ＋ | － | － |
| Rc190 | GE636562 | (ttc)_10_ | F:TGATTTATACTCTCAGTGCTGCTG  R:AAACGGGATCATCAATCAATTC | ＋(N) | － | － |
| Rc191 | EG694189 | (ctt)_11_ | F:GCTGCTTCACTTAAGCCTCTTT  R:TCTTCTCTCGAAACGAAGGAGT | ＋(N) | － | － |
| Rc192 | EG682410 | (ctt)_11_ | F:AGACCGACTGTTAGCATTAGCC  R:GAGGATTTCGGCTAAACTGTTG | － | － | － |
| Rc193 | EE258383 | (gaa)_11_ | F:CTACAAGAACAAAACGCCACCT  R:CGGACGACGAACTTTTAGATTC | ＋ | － | － |
| Rc194 | EG662514 | (tct)_11_ | F:CCTCCTGCTCTTGAGAACCTTA  R:GGAGCAAAAGCATTGAAAGAAG | ＋ | － | － |
| Rc195 | EG673660 | (ttc)_11_ | F:TATCTTTTGGACCTGTGGAAGG  R:AAGGCTGCTTAAGACTCGGTTA | ＋ | － | － |
| Rc196 | EG659581 | (tttg)_5_ | F:CCATTTGTTCTATTCCGTCTCC  R:CATTTCTGCCACTTTCTTCTCA | ＋(N) | － | － |
| Rc197 | EG667076 | (cttc)_6_ | F:TTCCAAGAACAAAAGACCCTTC  R:CTGCGACACATTCAAGATTAGC | ＋ | － | － |
| Rc198 | EE255935 | (ttta)_6_ | F:CACGAGGGAGAGAGAGAGAGAG  R:CCTCATTTCATTGCGTTATCAG | － | 🗸 | 🗸 |
| Rc199 | EE259200 | (aaga)_7_ | F:GGCACGAGGTGAGATTCTTATT  R:GGTTACTCTCAAAGGGTCGTTG | ＋(I) | － | 🗸 |
| Rc200 | EG670376s | (tctt)_7_ | F:ATGTATATGCAATCCCCACCTC  R:GGGAAGAGAGAATGGAGGTTCT | ＋(I) | － | 🗸 |
| Rc201 | GE636439 | (ct)_6_ | F:CACAGAGCTGAAGAAGCAAATG  R: AAAACCAAAGCCAGTAACTCCA | ＋(N) | － | 🗸 |
| Rc202 | GE636189 | (ct)_6_ | F:CTAGCTTAGCTGCGATCCCTAC  R: ATTGCTAAGGTGAGCTGGTGAT | － | － | － |
| Rc203 | GE635848 | (tc)_6_ | F:TCTGCTAACCAAACCCTTCAAT  R: TGGAGACAAAGAGCAAGAAACA | ＋(N) | 🗸 | 🗸 |
| Rc204 | GE634817 | (ag)_6_ | F:GCAATTCCATATTGGGTTTTGT  R: AGAAACTCAGGCAGGATCTCAG | ＋(N) | － | － |
| Rc205 | GE636325 | (ag)_6_ | F:TTCGTCCAAAACATTCAATGAG  R: AGCAATGTCTTGCACAGAGCTA | ＋(N) | － | － |
| Rc206 | GE634515 | (ct)_6_ | F:TGAGTTGTGTGTCACTCTTTGGT  R: CATCGGACGGTGCTAAAATAAT | ＋ | － | 🗸 |
| Rc207 | EG695167 | (tg)_6_ | F:GTTGCTCCAAAGGACAATAACC  R: GATGGACTGTTGATTCAGTGGA | ＋(N) | － | － |
| Rc208 | EG664289 | (at)_6_ | F:CGTTTAACAAAGAAGCAATCCA  R: ATGCCTTTGTCTTTCTCTTCCA | ＋(N) | 🗸 | 🗸 |
| Rc209 | EG660912 | (ct)_6_ | F:CTTCTTTTTGCCTCTCTGCAAT  R: CGTAAACCCTAATTGGGAAAGC | ＋(N) | 🗸 | 🗸 |
| Rc210 | EG661423 | (tg)_6_ | F:GTTGCTCCAAAGGACAATAACC  R: GATGGACTGTTGATTCAGTGGA | － | － | － |
| Rc211 | EE259858 | (ag)_6_ | F:TAGGGCAATTGGTGAAGAGAGT  R: TCTCACGTTCCTTCTCCTTTTC | ＋(N) | 🗸 | － |
| Rc212 | GE634557 | (tg)_7_ | F:CAAGTTGGATAACCGACCTACA  R: TGAATCACATTTCACACATCCA | － | － | － |
| Rc213 | EG699678 | (tg)_7_ | F:TGTTGTGCTTGCTTGAATTTTT  R: ACCCTAATTTCGCTTTCAATCC | － | 🗸 | 🗸 |
| Rc214 | EG697329 | (at)_7_ | F:CCTCGTGAGTCTCTCTTTCTCC  R: CTGTGTTGGCCTTTTCATCATA | － | － | － |
| Rc215 | EG694666 | (tc)_7_ | F:AGGAACTCCACAACTCACCCTA  R: CATCATCATCTTTGCATGCTTT | ＋(N) | － | － |
| Rc216 | EG692893 | (ag)_7_ | F:CCACCTAGCCACTCGATATTCT  R: CCCTCTTCTGTTTCTGGGTAGA | － | － | － |
| Rc217 | CF981379 | (at)_7_ | F:CTTCCCTTGCGTACTACGAGTT  R: GCTGTCTAAGCTTCCCAACTTT | ＋(N) | － | － |
| Rc218 | EG660996 | (tg)_7_ | F:TTGGATTGTCTCATGATTCTGC  R: AACGATCGTATACAGCAACCAA | －) | － | － |
| Rc219 | EE260711 | (at)_8_ | F:TACATGTGGCCATGCTCTTTAC  R: GCACTTGCAGTATTCCTTCGTT | ＋(N) | － | － |
| Rc220 | EE259493 | (tc)_8_ | F:AGAAGACGACGAAGAAAAGCAG  R: AGAAAACAAAGGGTATGGAGCA | ＋ | 🗸 | － |
| Rc221 | EE256035 | (ct)_8_ | F:AAATCGCTCCAGTTTTAACCAA  R: TCCAGAACTGCTGTGCTTTTTA | ＋(N) | 🗸 | 🗸 |
| Rc222 | EE257377 | (ga)_8_ | F:CTCACTCGTTGCAGAATTGACT  R: AGGGCGTAGGAAGGACACTTA | ＋(N) | 🗸 | 🗸 |
| Rc223 | GE634375 | (ta)_10_ | F:TAGAAAGAAAGCGACCCTTCTG  R: CACGATTGAAAGTTGAAACCAA | ＋ | 🗸 | － |
| Rc224 | EE255709 | (ta)_10_ | F:CAAAGATTCCAAGTAGCAAGCA  R: CAGCAGCAACCATATTACCAAA | ＋ | 🗸 | 🗸 |
| Rc225 | EG665471 | (ag)_10_ | F:CATAGCAAGGGGGTCACTAGAA  R: ACTGCTGAAGCTGCTGTCG | － | 🗸 | 🗸 |
| Rc226 | EG695020 | (ga)_10_ | F:CAAGACCAAAGAAACCGAAAAC  R: AGCGACACAAGTCAAGTCCATA | ＋ | － | － |
| Rc227 | EE254893 | (tg)_10_ | F:CCACTTTCCTCCTCCTTTCTCT  R: TCAACTGGGAGACAACCTCTTT | ＋(N) | 🗸 | 🗸 |
| Rc228 | EE254441 | (at)_11_ | F:CCGCCAAAAATACCAAGATTAT  R: ATTACTGCAAGTGCTCCCTACC | ＋ | － | － |
| Rc229 | EG672089 | (ta)_11_ | F:AGAACCGTGCAGTGGTCTTAGT  R: TTGCAGGATTGTGATAAGTGCT | ＋(N) | － | － |
| Rc230 | EE258412 | (ta)_11_ | F:AAATACCCGAACAAAAAGCTCA  R: CCTCTCCCATGGAGAAACTATG | － | － | － |
| Rc231 | EG693832 | (ga)_12_ | F:CTGGCATTTTCTCTTTCTGGAT  R: CCGTTGTATTTGCCATTCTTCT | ＋(N) | 🗸 | － |
| Rc232 | EG691859 | (ct)_12_ | F:TACCACCAACCGCCTTTAGTTA  R: TAAGAACACAGGTCTGCTGCAT | － | － | － |
| Rc233 | EG657403 | (ga)_12_ | F:TTGGTACACAACCTACATGAGC  R: CTCCTCCAACATTTCCTGGTAG | ＋ | － | － |
| Rc234 | EG669717 | (ta)_12_ | F:AATCCAGAGAGGCAAAACATTC  R: CACCATTGAAAGCCAGCTACTA | ＋ | － | － |
| Rc235 | EG667648 | (ta)_12_ | F:AATCAATCCAGAGAGGCAAAAC  R: CACCATTGAAAGCCAGCTACTA | － | － | 🗸 |
| Rc236 | EE260480 | (ct)_12_ | F:TTGGTTGGAAAAACAGATTCCT  R: AAACCCGCTTGAAATATGATTG | ＋ | 🗸 | 🗸 |
| Rc237 | EG697485 | (tc)_13_ | F:GCCACCATGTGAATTATCCTCT  R: TGTGAGAAGGAGAGGGTTTCAT | ＋(N) | － | － |
| Rc238 | EG691419 | (ct)_13_ | F:CTCTTTCCACCCTCCTCCTTAT  R: GTTGCTGTTGCCATTTCCTTAT | － | － | － |
| Rc239 | EG676447 | (tc)_13_ | F:CCAACAAATTCACTCCCTCTCT  R: CCAAGATTGAAGCAAAAACACA | ＋ | 🗸 | 🗸 |
| Rc240 | EG672885 | (tc)_13_ | F:GCCACCATGTGAATTATCCTCT  R: TGTGAGAAGGAGAGGGTTTCAT | ＋(N) | － | － |
| Rc241 | EE257541 | (ga)_13_ | F:TCACCCAAAATGTAAGAAAATGC  R: TGATTCTTCTGGGAAAGATGCT | － | － | － |
| RTc242 | EG695527 | (ct)_13_ | F:ACCCCTGCAAAACCCTAATAAT  R: TTCGGTGTAAAGAATCCGACTT | ＋ | － | 🗸 |
| Rc243 | EE254642 | (ta)_14_ | F:CAGAAGAAGCAGTAGCAGCAAA  R: CCCAAGTCCCAGATCTCTCTAA | ＋ | 🗸 | － |
| Rc244 | EG689468 | (ga)_14_ | F: CAACTTGTCTTTCTCTCCTCCA  R: CACGACCCAACAATGTATCATC | － | 🗸 | 🗸 |
| Rc245 | EG669444 | (ct)_14_ | F:TCACTTTTACCTCCCTCTGCTC  R: ATGGGTTTAGGAATGTTGTTGG | ＋ | 🗸 | 🗸 |
| Rc246 | EE259040 | (ag)_14_ | F:TGCAGTTTCGTCTCCTTCACTA  R: AATGACAATGGCGACTGATAAA | ＋ | － | － |
| Rc247 | EG680607 | (at)_15_ | F:AACAAAATCCAAACCCCAATC  R: CAGCATATGATTGTTCCTTCCA | ＋ | 🗸 | － |
| Rc248 | EG681668 | (at)_15_ | F:CAATCACCACCAAAAACCAAAG  R: CAGCATATGATTGTTCCTTCCA | ＋ | 🗸 | 🗸 |
| Rc249 | EG683395 | (ct)_15_ | F:GACTTCAACTTTCTACCTGACCTTG R: CTACCACCTGATTTCCCCATAA | ＋ | 🗸 | 🗸 |
| Rc250 | EG659605 | (tc)_15_ | F:AGCACTCTCAACTCCAACCTTC  R: CGGCCATAGTCTGTTTAGCTTT | ＋(N) | 🗸 | 🗸 |
| Rc251 | EG661770 | (ct)_15_ | F:AAACAAACAACACCGAAATCCT  R: CGTAGCGACTCATTTACACCAA | ＋ | 🗸 | 🗸 |
| Rc252 | GE634102 | (tttg)_5_ | F:CCATTTGTTCTATTCCGTCTCC  R: CATTTCTGCCACTTTCTTCTCA | ＋(N) | － | － |
| Rc253 | EG697485 | (aaag)_5_ | F:CATGAAACCCTCTCCTTCTCAC  R: ATCCGGTTCTTCTTCTTGTTCA | ＋(N) | 🗸 | 🗸 |
| Rc254 | EG659581 | (tttg)_5_ | F:CCATTTGTTCTATTCCGTCTCC  R: CATTTCTGCCACTTTCTTCTCA | － | － | － |
| Rc255 | EG683175 | (tttc)_5_ | F:CCGTAAGTCCCTGCGTTATTAT  R: CAAACGTCCAATACAAACCAAA | － | － | － |
| Rc256 | EG667814 | (agaa)_5_ | F:AGACAAACACATGCTGCCTTTA  R: ATGTGTGGTGGGGATGTTATCT | ＋ | － | － |
| Rc257 | EG672885 | (aaag)_5_ | F:CATGAAACCCTCTCCTTCTCAC  R: ATCCGGTTCTTCTTCTTGTTCA | － | 🗸 | 🗸 |
| Rc258 | EG662055 | (gaaa)_5_ | F:GCAGTTAGGCATCAAAATCCTC  R: GAAGTGCAGCTGTTAGGGTTTC | ＋ | 🗸 | 🗸 |
| Rc259 | EG692953 | (aaag)_5_ | F:ACGACTCCGACTCCTCTTACAC  R: TTTCTTCTTTAATCGGCGTCTC | ＋ | － | 🗸 |
| Rc260 | EG658641 | (tttc)_5_ | F:GAAGTGCAGCTGTTAGGGTTTC  R: GCAGTTAGGCATCAAAATCCTC | ＋ | 🗸 | 🗸 |
| Rc261 | EG661318 | (tttc)_5_ | F:ATCCTTTTTGGAATTTGGGATT  R: AATTTCCGGTATGAAGTAGCAGA | － | － | － |
| Rc262 | EG664709 | (aaag)_5_ | F:ACGACTCCGACTCCTCTTACAC  R: TTTCTTCTTTAATCGGCGTCTC | ＋ | － | 🗸 |
| Rc263 | EG670376 | (tctt)_7_ | F:ATGTATATGCAATCCCCACCTC  R: GGGAAGAGAGAATGGAGGTTCT | － | － | － |
| Rc264 | EE253962 | (aaga)_7_ | F:TGAAAGAGGAAACTCTGTTCCAG  R: GGTTACTCTCAAAGGGTCGTTG | ＋(N) | 🗸 | － |
| Rc265 | EG694178 | (aaga)_8_ | F:AAGAACACCCGCATAAAAGAAG  R: AAGCAGGCCTTTGTCAGTAGAC | － | － | － |
| Rc266 | EG668805 | (ttc)_12_ | F:TTTTCACTTGCACGATACCAAG  R: CGTTGAAACAACTCCTGAAATG | － | － | － |
| Rc267 | EV523911 | (ga)_5_ | F:GCAGCATTGTGAATGTAGAA  R: GGTAACCTCACAGCATCATT | － | － | － |
| Rc268 | EV523854 | (act)_4_ | F: TAGCCTCTCCCTTTCTCTCT  R: CTAATCTAACAGGCGGTGAC | ＋(N) | － | 🗸 |
| Rc269 | EV523854 | (tgc)_4_ | F:GTCACCGCCTGTTAGATTAG  R: GCTTACACCGTATCTTTTGG | － | － | － |
| Rc270 | EV523827 | (ctt)_8_ | F:CTCTTAAAACCCCCAATTTT  R: AATCTCTTTCAAATGCTGGA | － | － | － |
| Rc271 | EV523780 | (tga)_4_ | F:TTTGAGCCTGGTATTTTGAT  R: CGGGTTTAACTCATTCTTTG | ＋(I) | － | 🗸 |
| Rc272 | EV523864 | (gcg)_4_ | F:ACGATCTGCTTGTTCTCTGT  R: CAGGTCAAGATCTCCAACAT | ＋ | － | － |
| Rc273 | EV523749 | (caa)_4_ | F:TCTTAGTCTCCGCTTTCTTG  R: CTTTGGTCCATTGACGTATT | ＋(N) | － | － |
| Rc274 | EV523711 | (acc)_4_ | F:ACCACTACCGCTTCTACTCA  R: CAGCGAACTGAGTGTCTGTA | ＋(N) | － | － |
| Rc275 | EV523702 | (caa)_4_ | F:TAGCCTTTGCTTTCTTGTTC  R: CTTTGGTCCATTGACGTATT | ＋(N) | － | 🗸 |
| Rc276 | EV523696 | (tc)_11_ | F:TTTAGAATCGCTAGCAGACC  R: AGCAATAAAAACCAAGCAAG | － | － | － |
| Rc277 | EV523696 | (tag)_7_ | F:TGGGTTTTGATCCTTGTTAC  R: TGAGGCCTAATGTCTTCAAT | － | － | － |
| Rc278 | EV523693 | (tc)_5_ | F:TTCATCTTATGCTGCTGTTG  R: GAGGGCTTCTTTTCTTTTTC | ＋(N) | － | 🗸 |
| Rc279 | EV523665 | (gat)_8_ | F:CTGTGGTTGAGGATGAAGAT  R: GCTTTGCTTTGATCTACCAC | ＋(N) | 🗸 | 🗸 |
| Rc280 | EV523614 | (gac)_4_ | F:CGCATAAACCCTAAAGAAGA  R: TTTCATCATCTTCCTCATCC | ＋(I) | － | 🗸 |
| Rc281 | EV523587 | (gca)_4_ | F:AAGAAAGTGTATGCCCAAAA  R: AATTCCCTCTCCCATTGTAT | ＋(N) | － | 🗸 |
| Rc282 | EV523565 | (ctc)_4_ | F:CAAAAAGGCAAAGAAAGAAA  R: TCTCCAGAGTTGAGCAAAAT | ＋(N) | － | 🗸 |
| Rc283 | EV523554 | (cag)_5_ | F:GGCACTGATCAATTCAAGAT  R: CACTTGAGATCCACCAAGTT | ＋(N) | － | 🗸 |
| Rc284 | EV523549 | (aac)_6_ | F:ACACTAATCAAAGGCAAGGA  R: AAACCATGAGATCCAAGATG | ＋(N) | － | 🗸 |
| Rc285 | EV523511 | (ag)_10_ | F:AACACCACCTCAAATCAAAC  R: TCTTCTCACTGTTGTTGCTG | － | － | － |
| Rc286 | EV523484 | (agc)_4_ | F:GCAAAACCAGAAACATCTTC  R: TGTTGCATGGTGGAATAGTA | ＋(I) | － | 🗸 |
| Rc287 | EV523770 | (ctg)_5_ | F:CCTAAGGCAGCTAAGTCAAA  R: GCAACTGCTTATGCTTCTCT | ＋ | － | 🗸 |
| Rc288 | EV523478 | (taa)_4_ | F:TTCCAGCTCTTGTTCTTAGC  R: TTAATTGCTTTCTCCTCTGC | ＋(N) | － | 🗸 |
| Rc289 | EV523453 | (cac)_5_ | F:TTTTGCTATTGGACTTCGAT  R: TTGCAGGTGGAAGAACTACT | ＋(N) | － | 🗸 |
| Rc290 | EV523449 | (ggt)_5_ | F:ATCCAGAAAAGCGTGAGATA  R: GGAAAATATCGAATGGATCA | ＋(N) | － | 🗸 |
| Rc291 | EV523413 | (ctt)_6_ | F:TCTTCTTCGCTTTCAATTTC  R: TCACAGCATCATCTCCATAA | ＋(N) | － | － |
| Rc292 | EV523412 | (gcg)_5_ | F:GGTTTCGAATCTACAACTGC  R: CCTCTGACCTAAATGACAGC | ＋(N) | － | 🗸 |
| Rc293 | EV523368 | (cag)_5_ | F:TTGTCTCATGAAACCAATCA  R: TTCTGTTACAAGGGAGTGCT | ＋(N) | － | － |
| Rc294 | EV523693 | (tct)_5_ | F:GAATCCCAATGTCCAGTCTA  R: CCTGAAAGAGAACAAAGTGG | ＋ | － | 🗸 |
| Rc295 | EV523352 | (ttg)_4_ | F:AAGATTCGTGAGAAGAACCA  R: CAACATCCAGAAATGGACTT | ＋(N) | － | － |
| Rc296 | EV523311 | (gag)_4_ | F:CCTGGACGAGTAAAGAGAAA  R: CGAACCAAATATCTGACCAT | ＋(N) | － | 🗸 |
| Rc297 | EV523288 | (att)_5_ | F:TTCTGATTTTAGAGGCAAGC  R: TGAAAGAGCAGGAATTTGAT | ＋(I) | － | 🗸 |
| Rc298 | EV523252 | (ct)_5_ | F:AAGAGAGTTTGATCCAAGCA  R: TGTAAACGGGAAGAGGTTTA | ＋(N) | － | 🗸 |
| Rc299 | EV523221 | (cgc)_7_ | F:ATCGAACTCAAAATCGAAGA  R: TCTTGTGCGTGTGTATGTTT | ＋(N) | － | 🗸 |
| Rc300 | EV523208 | (gat)_5_ | F:TACTTGGTTTTCGCTATGGT  R: TACCAGAGACGGGTAAAGAA | ＋(N) | － | － |
| Rc301 | EV523200 | (tgg)_4_ | F:TACTTGGTCAAAGAGGTGCT  R: AGCATGATCAAGAAGAGGAA | ＋(I) | － | 🗸 |
| Rc302 | EV523171 | (tga)_4_ | F:TACTCGAATGAGCTCCTGAT  R: TCTTTTCAGAATCCGAGTGT | ＋(N) | － | － |
| Rc303 | EV523119 | (aat)_6_ | F:TTTGAATCTGGCTTCTGAAT  R: AGTTGAGCTTGTTTCGATGT | ＋(I) | － | 🗸 |
| Rc304 | EV523072 | (tc)_5_ | F:ACAAACGAGAACCAGAAGAA  R: TTGTTGATCAGTCAAGTCCA | ＋(N) | － | － |
| Rc305 | EV523046 | (aag)_5_ | F:GAGAGGGCTTATGATCCTTT  R: TGACAGTGATTTGTGAGCAT | － | － | － |
| Rc306 | EV523039 | (acc)_4_ | F:TCATGAAAGAAAAGGAAGGA  R: TGGCGTATTAGCCATTTAGT | ＋(N) | － | － |
| Rc307 | EV523024 | (tct)_4_ | F:GGTTTAGGATCAAGTGCAAG  R: AGAATCTGCGAACACAAAGT | － | － | － |
| Rc308 | EV523016 | (ag)_5_ | F:TTCCTCTTGAAAAACCAGAA  R: CTTTGGTCCATTGACGTATT | － | － | － |
| Rc309 | EV522971 | (gca)_4_ | F:TGTTCCTCTCTCTCTCCAAA  R: TGTTCAGCAGAACTCATACG | ＋(N) | － | － |
| Rc310 | EV522928 | (ga)_5_ | F:CTTCAAGCATAGACACCACA  R: TATACCTCGGATCCTGCTTA | ＋(N) | － | － |
| Rc311 | EV522890 | (gac)_4_ | F:GACTTAGGGAAGAAGGAGGA  R: CAACATCAACAACACCTACG | ＋(N) | － | 🗸 |
| Rc312 | EV521383 | (cga)_4_ | F:TTTTTCCTTCCGATTCTGTA  R: ATGACTCTTTCCCTTTCTCC | ＋(I) | － | 🗸 |
| Rc313 | EV521355 | (ac)_5_ | F:CACTCCCCTCAAACAAATAA  R: TGAGGGAGAAACAAAAAGAA | ＋(N) | － | － |
| Rc314 | EV521344 | (aag)_5_ | F:GAATGATGGAGAAAGGTCAA  R: TCCCATCTATCAGGCAATAC | ＋(N) | － | － |
| Rc315 | EV521242 | (gaa)_4_ | F:TCTACCCACTTGTGTAATTGAT  R: TTATTAATGCTATCCTCCTCCT | ＋(N) | － | － |
| Rc316 | EV521221 | (ctt)_4_ | F:ACCCATATTACTTGCTTCACT  R: CTCTGTGGAACTGTGAACTTA | ＋(N) | － | － |
| Rc317 | EV523589 | (aag)_4_ | F:CGCCAAGCTTATAAGAGAAA  R: GAGTCTGAAGGGAGAGGAAT | ＋ | － | 🗸 |
| Rc318 | EV521221 | (atc)_6_ | F:GAGAGGCAAGATTATGCAAC  R: ATCAGCAAAAAGGTAGTCCA | － | － | － |
| Rc319 | EV521182 | (tga)_4_ | F:AGGTTGCTGATGAGTTTGATA  R: AAATAAGCCGTTTCTTTGTTT | ＋(N) | － | － |
| Rc320 | EV521168 | (gtat)_5_ | F:TTCCTTTTTCATGCAGTAAATC  R: GAGTTCTTCATTAAGCCATCAA | ＋(N) | － | 🗸 |
| Rc321 | EV521164 | (ctg)_5_ | F:ACATCAGAGAGCTCAGCATT  R: AAAACCAGCCACCAATAATA | ＋(N) | － | 🗸 |
| Rc322 | EV521159 | (gaa)_4_ | F:TTTTAGTATTTTGCCCTGAA  R: AACAGAGGGAAGAGAATGAT | ＋(N) | － | 🗸 |
| Rc323 | EV521151 | (atg)_5_ | F:CTTGCAGAGTAAGGTCAAAG  R: CAATAACAAGACCCGTTTTA | ＋(N) | － | － |
| Rc324 | EV521144 | (ag)_5_ | F:TAACAAGATTGCTGGGTTTT  R: GAAACTTCAGGCACAAAGTC | ＋(N) | － | － |
| Rc325 | EV523460 | (tc)_5_ | F:ATCACCCACAATTACCCATA  R: CGAGAGAACGAAAAGAGAAA | ＋ | － | 🗸 |
| Rc326 | EV521121 | (gaa)_4_ | F:TGCAGGTATCAAGGTGTACT  R: TTGTACTGATCACCTCACCT | ＋(N) | － | － |
| Rc327 | EV521119 | (gaa)_4_ | F:TGCGTTGCTTGATTTTATTA  R: ATCCTCTACGACGATGCTTA | ＋(N) | 🗸 | 🗸 |
| Rc328 | EV523432 | (tc)_12_ | F:GCAGAAAGAACACGAATCTC  R: AGCAATAAAAACCAAGCAAG | ＋ | － | 🗸 |
| Rc329 | EV521100 | (cac)_4_ | F:TTTCACCGAAAAGAGTTCAA  R: GAACAGTTCTCCTCCTCCAC | ＋(N) | － | 🗸 |
| Rc330 | EV521091 | (acg)_5_ | F:AATGAACACTCCAAATCCAG  R: CATCATCATCATCTTCATCG | ＋(N) | － | － |
| Rc331 | EV523389 | (gag)_4_ | F:TTAGCTGCTTCGATTTCTTC  R: CAACATTAGCAGGCTGTGTA | ＋ | － | － |
| Rc332 | EV521091 | (gat)_5_ | F:TATAGTCGACGAAGTTACCG  R: AAAGTTCACCTGTATGACCA | ＋(I) | － | － |
| Rc333 | EV521088 | (gtat)_5_ | F:ATTTCCTTTTTCATGCAGTA  R: GTAACAAGAAAGGGGAGTTC | ＋(N) | － | － |
| Rc334 | EV521086 | (tc)_9_ | F:CACTAAACCTTTAAACACACG  R: TGAGAAAAGATACAAGCTTCA | ＋(N) | － | 🗸 |
| Rc335 | EV521076 | (gaa)_4_ | F:AAATCAACACATCTCTGGAA  R: GGCTTCTCTTCACTTCTCTT | ＋(N) | － | 🗸 |
| Rc336 | EV521068 | (tc)_5_ | F:CCTATTAGCCCTCAAATCTT  R: GTTGAAAGCAATTCACTCAT | ＋(N) | － | － |
| Rc337 | EV521039 | (atg)_4_ | F:TTGAAGAAGAGATTGGAGAAGT  R: AACATTATAGCCTTGCTGTCTT | ＋(N) | － | － |
| Rc338 | EV521017 | (ag)_18_ | F:CAGTCTTTTGTGTTTAGGAAGA  R: TGTATATTCAACCCATGTTCTC | ＋(N) | － | － |
| Rc339 | EV521011 | (ag)_10_ | F:AAATCAAACACCACACCTTA  R: CTCCCATTCTTAAGCTCTTT | ＋(N) | － | － |
| Rc340 | EV520990 | (tct)_4_ | F:GTTCTGACGAGAAGGCAAAT  R: TTTTATGTCCAAACCCCTTC | ＋(I) | － | － |
| Rc341 | EV520988 | (tct)_7_ | F:GTTGTTTGTCTGCATTTTTCTA  R: TCTCTTAAACTGGAAAGCTAGG | ＋(N) | － | － |
| Rc342 | EV520982 | (gaa)_5_ | F:GGTATAGAAGAAGACGAAGACG  R: AAAATCTACCCATTTCAACAAC | ＋(N) | － | － |
| Rc343 | EV520975 | (gtat)_5_ | F:CATGATAATGATTTCCTTTTTC  R: AAGAGCTCACTCCTTACATAGA | ＋(N) | － | － |
| Rc344 | EV520939 | (ag)_5_ | F:TTCCCAAGAAAGAGAAAGAGTA  R: AAGGTAGTGTGGATAAAAGCAC | ＋(N) | － | － |
| Rc345 | EV520909 | (tag)_7_ | F:GAGATTGGAATTTCTTCATTTA  R: CTTCGTAGAACAATGATTTCTT | ＋(N) | － | － |
| Rc346 | EV520890 | (tac)_4_ | F:ACCAAGAAAGAAAGACGACA  R: AAAGAGCAGACAAAAAGTGC | ＋(N) | － | － |
| Rc347 | EV520879 | (gca)_4_ | F:TCTCTCTATCAAACGACTGC  R: AAGAAGGGTCAAAACTCATT | ＋(N) | － | － |
| Rc348 | EV520871 | (tctt)_4_ | F:TTTATGTGTCAAATGTTGTTGC  R: TTTTCTTTTCTATTGCACGAGT | ＋(N) | － | － |
| Rc349 | EV520857 | (ttc)_4_ | F:CTATTGGCATTTCAAGGAAG  R: TCACAAACACAAGGAGAACA | ＋(N) | － | 🗸 |
| Rc350 | EV520852 | (tct)_4_ | F:GGTTTAGGATCAAGTGCAAG  R: ACGCTTCTGTTCCAAAATTA | ＋(N) | － | － |
| Rc351 | EV520822 | (gag)_5_ | F:GAAAGCAAAGAAGAAAGGAA  R: CCAGAAAACTTAATCGGTTC | ＋(N) | － | － |
| Rc352 | EV520724 | (tca)_4_ | F:GTGTCGGTCCTGATGATACT  R: CTTTTCTAATGCGTTTGGAT | ＋(N) | － | － |
| Rc353 | EV522755 | (gag)_4_ | F:TCCTGGACGAGTAAAGAGAA  R: AACGCACAAACAAAATACCT | ＋(N) | － | － |
| Rc354 | EV522661 | (gct)_4_ | F:ATTCAATGCTCTCTCCAGAA  R: CAGGAAAGAAATCTCCAGTG | ＋(N) | － | － |
| Rc355 | EV522623 | (agg)_4_ | F:ATTTGTGCTATGGAGTCACC  R: CATCAGTTTTCACAACATGC | ＋(N) | 🗸 | 🗸 |
| Rc356 | EV522607 | (ga)_5_ | F:CAAATTCCCAATTCTGTTGT  R: CTCGCATTTCTTTAGCTGTT | ＋(N) | － | 🗸 |
| Rc357 | EV521100 | (gaa)_6_ | F:ATGGAGTTTGAGGATCAAGA  R: AACTCGCTGTCATTTTGACT | ＋ | － | 🗸 |
| Rc358 | EV521100 | (cag)_6_ | F:AGTCAAAATGACAGCGAGTT  R: CGTGGTTCTTTAAGCACTCT | ＋ | － | 🗸 |
| Rc359 | EV522477 | (aat)_6_ | F:TTTGAATCTGGCTTCTGAAT  R:AGTTGAGCTTGTTTCGATGT | ＋(N) | － | － |
| Rc360 | EV522555 | (aga)_4_ | F:TAGAGAAAATGTTGGCGAGT  R: TAACGTTGTTTTCCGAAGTT | ＋(N) | － | － |
| Rc361 | EV522475 | (tc)_11_ | F:GCAGAAAGAACACGAATCTC  R: AGCAATAAAAACCAAGCAAG | ＋(N) | － | － |
| Rc362 | EE254005 | (at)_7_ | F:TGCTGGCTTCTTTATCCTTGA  R: AATTCGGCACGAGGAATATG | － | － | － |
| Rc363 | EG695457 | (ct)_7_ | F:GCTACCTCCCTCTACCTTTCG  R:CTTTGGATGCTGCCATTTTT | ＋(I) | － | － |
| Rc364 | EG660783 | (tga)_7_ | F:ATTCAGCTAGCGGGAACTCA  R: TTGGTGTCTCCTCATCCTCAC | － | － | － |
| Rc365 | EV520933 | (taa)_6_ | F:CCATCGCTTGTCTAGTGTTT  R: CATTACCCAAAGAAGACTCG | ＋(I) | － | － |
| Rc366 | EG699678 | (tg)_7_ | F: AGGGAATTTGTTGTGCTTGC  R: CCCAAAAACCGAACCCTAAT | ＋(N) | 🗸 | 🗸 |
| Rc367 | EG660671 | (gca)_7_ | F: CCTGTTGCACCTTACCCTGT  R: AGCTTGAGGTGTGTGCCTCT | ＋(N) | － | － |
| Rc368 | EG694666 | (tc)_7_ | F: CTGCATGCATTTGGCTTCTA  R: GATGTGGTGTTTCGGTGATG | ＋(N) | － | 🗸 |
| Rc369 | EG659273 | (ta)_7_ | F: TTGAGCATTCCTCGGTGATA  R: ATGTCGGCGTCTCCTCTAAA | ＋(N) | － | 🗸 |
| Rc370 | EG657164 | (cga)_7_ | F: TGGACATGGTATGGAACACG  R: TTTTCTTCATGCTGCCCTTC | － | － | － |
| Rc371 | EG659634 | (gaa)_7_ | F: CTGCAGAACCTGGGCTAGAG  R: GGGTTTGATGGGTTCAATCTT | ＋(N) | 🗸 | 🗸 |
| Rc372 | EG660686 | (aga)_7_ | F: AATCTCCCCCAATCAATTCC  R: AGCTGCTTCATCGAGGTTGT | ＋(N) | － | 🗸 |
| Rc373 | EG659545 | (acc)_7_ | F: GCCATCTCGTTTCACCATCT  R: ATTCGGAGGGAGCTGTTCTT | ＋(N) | － | － |
| Rc374 | EV520791 | (gct)_5_ | F:GATCTCGTAATGAATGTTGG  R: CTCTCTTCTTGAGGCTCTTC | ＋ | － | － |
| Rc375 | CF981374 | (at)_8_ | F: TGATGTCTGTTCCCTCTGCTT  R: TCATGGTTGCCCGTATACAA | － | － | － |
| Rc376 | EG690964 | (ct)_8_ | F: GGGGTGTCGCTTCAAAGATA  R: GGGCAGTACAACGAGGAATG | ＋(N) | 🗸 | － |
| Rc377 | EG689942 | (gat)_7_ | F: CCCGAATCAAAAACCAAAAA  R: TCCAAATGGAAATGGTACGG | ＋(N) | 🗸 | 🗸 |
| Rc378 | EG657435 | (gt)_9_ | F: CCTCGCGTGAATCGTACTTT  R: TCGCTGTTTGTGTTTGTCGT | － | － | － |
| Rc379 | EG660663 | (gt)_9_ | F: CAAGGACAAAAGAATTTGGACA  R: CGATCGCTGTTTGTGTTTGT | － | － | － |

Note:

a), ＋(N) denotes that PCR produces monomorphic amplicons;

b), ＋ denotes that PCR produces polymorphic amplicons;

c), ＋(I) denotes that the PCR amplicons contain obvious introns, excluding from analyses;

d), － denotes that PCR fails;

e), 🗸 denotes that the successful transfer of markers is identified in *Jatropha* *curcas,* or *Speranskia cantonensis*.
