# Supplementary material for: Exploiting EST databases for the development and characterization of EST-SSR markers in castor bean (Ricinus communis L.)
Source: BMC Plant Biol. 2010 Dec 16;10:278. doi: 10.1186/1471-2229-10-278 (PMC3017068; doi:10.1186/1471-2229-10-278)
Supplement: Additional file 2 — Table S2: Validation and characterization of polymorphic SSR markers derived EST sequences.doc [file 1471-2229-10-278-S2.DOC]

**Table S2: Validation and characterization of polymorphic SSR markers derived EST sequences**

| **EST ID** | **Code** | **Forward primer (5’-3’)** | **Reverse Primer (5’-3’)** | **Repeat** | **Expected Size of Allele (bp)** | **Allele Size**  **Range (bp)** | **No. of Alleles** | ***He*** | **PIC** |
| --- | --- | --- | --- | --- | --- | --- | --- | --- | --- |
| EG662973 | Rc02 | CCTCCATTGGTTTCTCCATTTA | TGATTCGAGATCAGAGTTGACG | (GT)_9_ | 269 | 256-271 | 4 | 0.354 | 0.386 |
| EG686206 | Rc03 | TGATGCTAATGGCCTACTCAGA | TAATAGCACCACCATCAACTGC | (AT)_8_ | 264 | 260-268 | 3 | 0.226 | 0.212 |
| EG682908 | Rc04 | TGAAAGCCACACAATAGTCCTG | TGAGATTCATCTTGTGGAGGTG | (CT)_8_ | 190 | 186-190 | 2 | 0.278 | 0.239 |
| EE255832 | Rc05 | CATCACCTCTATCCATCCGTTT | CCTTCATCATTGAACTCCCTTC | (AC)_8_ | 200 | 200-227 | 4 | 0.709 | 0.658 |
| EG663122 | Rc07 | GGAAGAAGAGAAACAGCAAGGA | GTGGAGAGCCTCGACTAAGAAG | (GA)_9_ | 241 | 234-247 | 6 | 0.695 | 0.658 |
| EG686478 | Rc09 | GTCCAGGTTCACCATCTTGATT | ACCACCGTAGAAAGTAGCATGG | (CT)_9_ | 280 | 280-288 | 3 | 0.226 | 0.212 |
| EG678906 | Rc10 | CAGAAGAGCTCATGTGTTGTGG | AGTCTTGTGATCCTCACACTGC | (CA)_9_ | 241 | 235-243 | 3 | 0.156 | 0.151 |
| GE634375 | Rc11 | TAGAAAGAAAGCGACCCTTCTG | AAGAAGGAGGAGGAGGAGAAA | (TA)_10_ | 242 | 238-268 | 4 | 0.581 | 0.539 |
| T14845 | Rc12 | ACATTATAGCGAGTGTGCTTGC | TGGACTTCTCAAAGCTGCATTA | (AC)_11_ | 232 | 232-262 | 6 | 0.764 | 0.729 |
| EG666688 | Rc14 | TTCTGATGACATTCCCAGAAAC | AGATATCTGGGGAGACGAAGC | (TA)_13_ | 224 | 214-227 | 4 | 0.489 | 0.429 |
| EE259292 | Rc16 | CATGAACCCCACAATTTACAGA | GACAGACCCATCTCAAGATTCC | (TC)_15_ | 254 | 242-252 | 4 | 0.325 | 0.305 |
| EG674726 | Rc17 | CCTCCCAAATCATAGTCCATTG | GATCAAGGTGATTAGGGACCAA | (AT)_16_ | 255 | 249-261 | 4 | 0.503 | 0.456 |
| EG670576 | Rc19 | TCGTCCTCCCTGCTTTTATTTA | GCACCAAATCTTTTTAGGCAAG | (AT)_18_ | 192 | 278-296 | 6 | 0.521 | 0.490 |
| EE259262 | Rc20 | CGTTTAAGGATCCGCATATCTC | GCTCAACCACCTCAAGATTTTC | (AG)_20_ | 244 | 254-260 | 2 | 0.375 | 0.305 |
| EE256592 | Rc22 | ATGAAGTTCCAGCGTAACCATT | ATCTGAAACCATGGGTCATCTC | (AT)_22_ | 254 | 272-294 | 4 | 0.573 | 0.483 |
| EG662293 | Rc23 | GCCGTCCTATTTCTCTCCTTCT | TTTCTACATCGGCAGTGAAAGA | (TC)_22_ | 208 | 249-255 | 3 | 0.288 | 0.264 |
| EE255894 | Rc24 | GAGAGCGTGGCTTCTCTACTTC | CAGTTTCACACCAAGCTGGTAA | (AT)_23_ | 233 | 259-273 | 3 | 0.594 | 0.523 |
| GE633816 | Rc25 | ACCCCTGCAAAACCCTAATAAT | TTCGGTGTAAAGAATCCGACTT | (CT)_23_ | 259 | 259-265 | 3 | 0.642 | 0.570 |
| EG664176 | Rc26 | CACTTCTTCCTGATGCTCTCCT | AGGGGTGAAGGGATTTGTTTAT | (AT)_27_ | 197 | 191-215 | 5 | 0.361 | 0.346 |
| EG684662 | Rc27 | AGATCTTGGAGGCTGTTGTCAT | CTGCTTCAGGATCTCGAAAAGT | (AT)_28_ | 243 | 143-257 | 2 | 0.444 | 0.346 |
| EE260610 | Rc28 | CCCAAGTCCCAGATCTCTCTAA | CAGAAGAAGCAGTAGCAGCAA | (AT)_29_ | 233 | 223-237 | 3 | 0.663 | 0.589 |
| EE257519 | Rc29 | ACGAGGCTCAGACTTGTGTCTA | GCTGCTAATTGGCTGAAATTG | (AT)_32_ | 264 | 250-266 | 4 | 0.538 | 0.482 |
| GE632365 | Rc31 | GCAGCTAGCCTTATTGAGGAAA | GAAAATATCTCCACCACCTTGC | (TA)_11_ | 174 | 174-176 | 2 | 0.486 | 0.368 |
| EE254685 | Rc32 | CAGTTTCACACCAAGCTGGTAA | GTTAAGGCAGGTAACGATACGG | (TA)_23_ | 259 | 259-265 | 2 | 0.375 | 0.305 |
| GE632805 | Rc33 | CTTGGCAGGTCACAAAGAACTA | GCCGTCCAGAATTGATAGGTAG | (TC)_10_ | 233 | 233-237 | 2 | 0.330 | 0.275 |
| EE260187 | Rc35 | AAGCGAATTCCATACTCCTTCA | AAACCCACGTCTATCCTCAAAA | (AG)_13_ | 238 | 238-241 | 2 | 0.153 | 0.141 |
| EE254189 | Rc40 | GCATCATCACAGCCTCAAATTA | TATCTGAGCCTCCTCTTTCCAG | (AG)_15_ | 163 | 163-184 | 3 | 0.642 | 0.570 |
| EG698508 | Rc45 | TATTACAATACCGCCCCTATGC | ACCGCTGTTATTCCTGTCAAAT | (CAC)_6_ | 280 | 280-284 | 2 | 0.219 | 0.195 |
| EG664367 | Rc51 | AGGACTTATTCGACCCAGATGA | GATGAGTACTGAAGCGATGGTG | (GCC)_6_ | 207 | 260-266 | 2 | 0.278 | 0.239 |
|  |  |  |  |  |  |  |  |  |  |
| EG697581 | Rc53 | TGCAAGAACAAGATTGAGTCCA | GGCTCCAACAATAACAAAGGAG | (TGC)_6_ | 260 | 252-260 | 2 | 0.080 | 0.077 |
| EG689318 | Rc57 | TGTAGCAGAGAAATGCAAGGAA | ATTTAAGGTCAGCCCAAGAACA | (ATC)_7_ | 207 | 225-231 | 3 | 0.288 | 0.264 |
| EG662586 | Rc61 | GCACTGAGGGTTAATTCTGGAC | GTCGTAGACCACCTTAGCATCC | (GGC)_7_ | 232 | 205-211 | 2 | 0.492 | 0.371 |
| EG701975 | Rc65 | CGTAGCTGAAGCTGAACAAAGA | GCTTGACGGAGAGTAGAGCAAT | (AGA)_8_ | 261 | 271-286 | 3 | 0.508 | 0.415 |
| GE633761 | Rc73 | AGCCTGAATCCTCTGATGTAGC | GGTCCTGGTTCAGGATCAGTAA | (CCA)_9_ | 178 | 178-184 | 2 | 0.486 | 0.368 |
| EG698249 | Rc74 | CTGAAGTTCCAGAGCTTCCTGT | TGAGAGGAAGGATTACGGAGAC | (CTG)_9_ | 200 | 200-208 | 2 | 0.469 | 0.359 |
| EG696213 | Rc76 | GGAGGGATATGGATTTCAGATG | CGGAAGATGAGAGAACAGGTT | (ATA)_9_ | 225 | 223-230 | 3 | 0.431 | 0.392 |
| EG695475 | Rc84 | ACCTCTCCTTCCCTCATCTCTC | GAATCTAACGGTGCTGATCCTC | (AAG)_11_ | 184 | 190-200 | 3 | 0.471 | 0.378 |
| EG660729 | Rc85 | GCCACTATGGTGTCATCTCGTA | CTTTTAATTTTAGGCGGTGGAG | (CAC)_11_ | 165 | 241-291 | 3 | 0.572 | 0.509 |
| EG681190 | Rc86 | GCAACTTCAGATGCAACAACTC | TCATACATCTTTGTTGCCAAGG | (CAG)_11_ | 245 | 275-284 | 2 | 0.153 | 0.141 |
| EG687367 | Rc87 | GCCACTTCCGATTATTTCTTTG | CGGTTCACACCCATCTCTTTAT | (TAA)_11_ | 275 | 260-268 | 2 | 0.187 | 0.169 |
| EG667050 | Rc88 | CACCAATCTCATCACCAACACT | GAATTGTGGGGTTTAGGGTTT | (TCT)_11_ | 246 | 252-264 | 3 | 0.344 | 0.307 |
| EG679278 | Rc89 | TCTGTTAAACAACGGGAAAACC | CTACAAGAACAAAACGCCACCT | (TTC)_11_ | 261 | 252-261 | 2 | 0.249 | 0.218 |
| EG666840 | Rc90 | CACCTCCACAAGAACAAGAACA | CGGACGACGAACTTTTAGATTC | (GAA)_11_ | 245 | 233-245 | 2 | 0.249 | 0.218 |
| EG665468 | Rc91 | AGTGGCATGGGCATATTATGTT | AACTCTCTAAGCCCCAAATTCC | (GAG)_12_ | 219 | 219-225 | 3 | 0.601 | 0.533 |
| EG691818 | Rc94 | ACGCCACAAACACACATATCAT | CAAGTGCATCTGAAACGACAAT | (GAA)_15_ | 248 | 231-248 | 3 | 0.601 | 0.516 |
| EG699176 | Rc95 | TAGACCCAGCAGTGCTCATAAA | CCACCTTTAGGTTGGATGTTGT | (CAA)_19_ | 235 | 211-241 | 3 | 0.594 | 0.511 |
| EG693205 | Rc96 | ATGGCAACTGTTGAGGTAATCC | CTACATGGCAGCACCAAGAAT | (AAAG)_6_ | 275 | 272-278 | 3 | 0.351 | 0.322 |
| EG681785 | Rc100 | CCAAGAAAACAACTCCGGTTAC | GAGGACATGGGTTGTTGTTGAT | (CAAT)_6_ | 180 | 180-184 | 2 | 0.153 | 0.141 |
| EG678636 | Rc103 | CCAGAAATGCATCTGGATCG | TGGACTGGAGATGCTGATTATG | (TA)_7_ | 241 | 235-241 | 2 | 0.486 | 0.368 |
| EG686206 | Rc104 | TGATGCTAATGGCCTACTCAGA | TAATAGCACCACCATCAACTGC | (AT)_8_ | 262 | 262-266 | 2 | 0.353 | 0.291 |
| EE254243 | Rc112 | TATCTGAGCCTCCTCTTTCCAG | GGAACGACAAACACCACAACTA | (TA)_10_ | 265 | 265-283 | 4 | 0.601 | 0.524 |
| EG679802 | Rc113 | TCAGTTGAACTCAAGGTCAGTCA | AAGCAGAGTGCTTCTTCAATCC | (AG)_11_ | 228 | 228-234 | 2 | 0.330 | 0.275 |
| EG689244 | Rc116 | TCCTTTCAGACCGAAGGAGATA | ATAACCATAACCCCATCCATCA | (AG)_12_ | 252 | 250-261 | 3 | 0.254 | 0.231 |
| EG691558 | Rc117 | CGTTTCCCTACGCTTTCTACAT | TGATTCAGGTCCCATATTAGCC | (AT)_12_ | 232 | 222-238 | 3 | 0.559 | 0.470 |
| EG663853 | Rc119 | CAAACTAATCACACGCTTTCCA | ATTCTTCGTCGTCTTCTTTTGC | (AG)_13_ | 189 | 195-203 | 2 | 0.486 | 0.368 |
| EG697485 | Rc121 | GCCACCATGTGAATTATCCTCT | TGTGAGAAGGAGAGGGTTTCAT | (TC)_13_ | 245 | 248-250 | 2 | 0.219 | 0.195 |
| EG669444 | Rc122 | CTGTCAAAACATTCTTCGCATC | AATGTTGTTGGGATGAATAG | (CT)_14_ | 266 | 262-274 | 6 | 0.469 | 0.439 |
| EE256105 | Rc124 | GACAGACCCATCTCAAGATTCC | CATGAACCCCACAATTTACAGA | (AG)_15_ | 254 | 243-254 | 3 | 0.226 | 0.212 |
| GE632416 | Rc126 | CAGTATCACGCTGTGGTTGTTT | CTTTGCCGACTGGAATATCAAC | (TG)_15_ | 180 | 184-186 | 2 | 0.492 | 0.371 |
| EG689641 | Rc128 | TTGGGGACTATGATCAAACTGA | TTTAAACTGCCATCAACGACAC | (TA)_16_ | 161 | 157-169 | 3 | 0.403 | 0.363 |
| GE634251 | Rc129 | TACTGCAACTCAATCCACTGCT | GATAGTGCCTTTGCCTCTTTTC | (TC)_16_ | 247 | 241-253 | 4 | 0.580 | 0.523 |
| EG692156 | Rc130 | TGAACTCACAATCAGTCCCTTC | TCTGGTACAGGTGATGATTTCG | (CT)_17_ | 229 | 227-233 | 3 | 0.403 | 0.363 |
| EG685152 | Rc131 | GATCATTTGCATGGCTGAAGTA | CCATAGCTTTGGACACACTCAC | (TA)_17_ | 180 | 168-180 | 3 | 0.344 | 0.307 |
| EG694164 | Rc132 | TCTTCACAGCTATTGGCAACAC | GGAAGCTAGGGATTAGGGATTG | (TC)_17_ | 189 | 183-189 | 2 | 0.330 | 0.275 |
|  |  |  |  |  |  |  |  |  |  |
| GE634416 | Rc135 | CTGCTTCTCTCTGCATTGTGTA | CCATTCCTTCTCTGGTTTCTTG | (GA)_19_ | 172 | 168-180 | 4 | 0.385 | 0.359 |
| EG680179 | Rc138 | CTTCCCAACAGCTCAATTTCTT | CACATGCATACATAAACCAGCA | (TC)_21_ | 269 | 265-269 | 2 | 0.413 | 0.328 |
| EE254161 | Rc139 | AGGTAACGATACGGTGTTGGAG | CAGTTTCACACCAAGCTGGTAA | (AT)_23_ | 225 | 213-231 | 5 | 0.733 | 0.687 |
| EE259204 | Rc140 | TCTAAGATGGTCTCGCCTCATT | GACCCATATAGGGCAAGGTTTA | (AT)_24_ | 179 | 163-183 | 6 | 0.780 | 0.748 |
| EG700448 | Rc141 | AATTGCCAGCCAAACATAAGAT | TAAATTGGGTTAATGGGTTTGG | (AG)_25_ | 176 | 176-190 | 3 | 0.351 | 0.322 |
| EG693883 | Rc158 | CCCCGACGATAACTACCATAAA | AGGTCATCGAATAACGACCAAG | (ACC)_7_ | 224 | 154-166 | 3 | 0.536 | 0.430 |
| GE635881 | Rc165 | ATTCCTTTCACACACGCTCTCT | GTATTTTAGGGGAATGGGGTTG | (GCA)_7_ | 249 | 240-249 | 3 | 0.468 | 0.411 |
| EG684973 | Rc166 | AGTTGCAGTTGAGGTTGTGATG | AAGGCCTCAGTTAACCAATGAA | (GGT)_7_ | 185 | 171-177 | 2 | 0.187 | 0.169 |
| GE632520 | Rc167 | GTCTGTGACAACAACCACGAAC | GAGATGGGAGAGATGAAGAGGA | (TTC)_7_ | 167 | 171-184 | 2 | 0.353 | 0.291 |
| GE633169 | Rc168 | TTCTTCCTCCTCCTCCACATAA | GAGGAGAAATGAAGCATTACGG | (AGA)_8_ | 213 | 214-219 | 2 | 0.187 | 0.169 |
| GE633829 | Rc176 | TACCCAATGACTGCAATCTTCA | GGGTTTGTTGAGAAGGAGAAAA | (CTT)_8_ | 222 | 219-230 | 3 | 0.617 | 0.540 |
| GE635438 | Rc182 | GCCAATCTGTTCACATACCTCA | GTCGAACTTTCATACCGGAAAA | (TCT)_9_ | 170 | 168-180 | 4 | 0.621 | 0.569 |
| EG671871 | Rc184 | CGCATCTTGCATTTCTTTTGT | AGGATGGCCTGTGTCTAATAGC | (ATA)_10_ | 246 | 241-246 | 3 | 0.448 | 0.397 |
| EG683300 | Rc186 | AGATCAAATCGGGGAAGAGAAT | CCATCATCAGTAGGTGGCATTA | (GGA)_10_ | 175 | 172-178 | 2 | 0.492 | 0.371 |
| EG662044 | Rc188 | TCTTCTTCGCCTCCAATAAAAC | TGTAGGGACAAAGCGATTCATA | (TTC)_10_ | 157 | 157-160 | 2 | 0.278 | 0.239 |
| EG666688 | Rc189 | CCAGAAATGCATCTGGATCG | TGGACTGGAGATGCTGATTATG | (TTC)_10_ | 241 | 235-241 | 2 | 0.469 | 0.359 |
| EE258383 | Rc193 | CTACAAGAACAAAACGCCACCT | CGGACGACGAACTTTTAGATTC | (GAA)_11_ | 227 | 233-239 | 2 | 0.249 | 0.218 |
| EG662514 | Rc194 | CCTCCTGCTCTTGAGAACCTTA | GGAGCAAAAGCATTGAAAGAAG | (TCT)_11_ | 249 | 249-252 | 2 | 0.08 | 0.077 |
| EG673660 | Rc195 | TATCTTTTGGACCTGTGGAAGG | AAGGCTGCTTAAGACTCGGTTA | (TTC)_11_ | 222 | 219-225 | 3 | 0.469 | 0.391 |
| EG667076 | Rc197 | TTCCAAGAACAAAAGACCCTTC | CTGCGACACATTCAAGATTAGC | (CTTC)_6_ | 211 | 207-213 | 3 | 0.508 | 0.415 |
| GE634515 | Rc206 | TGAGTTGTGTGTCACTCTTTGGT | CATCGGACGGTGCTAAAATAAT | (CT)_6_ | 193 | 193-228 | 2 | 0.375 | 0.305 |
| EE259493 | Rc220 | GTTGCTCCAAAGGACAATAACC | GATGGACTGTTGATTCAGTGGA | (TG)_6_ | 180 | 182-184 | 2 | 0.413 | 0.328 |
| EE255709 | Rc223 | TAGAAAGAAAGCGACCCTTCTG | CACGATTGAAAGTTGAAACCAA | (TA)_10_ | 189 | 189-211 | 6 | 0.551 | 0.524 |
| EG695020 | Rc224 | CAAAGATTCCAAGTAGCAAGCA | CAGCAGCAACCATATTACCAAA | (GA)_10_ | 246 | 244-252 | 3 | 0.156 | 0.151 |
| EE254893 | Rc226 | CAAGACCAAAGAAACCGAAAAC | AGCGACACAAGTCAAGTCCATA | (GT)_10_ | 243 | 239-243 | 2 | 0.219 | 0.195 |
| EE254441 | Rc228 | CCGCCAAAAATACCAAGATTAT | ATTACTGCAAGTGCTCCCTACC | (AT)_11_ | 236 | 232-236 | 2 | 0.153 | 0.141 |
| EG657403 | Rc233 | TTGGTACACAACCTACATGAGC | CTCCTCCAACATTTCCTGGTAG | (GA)_12_ | 233 | 229-233 | 3 | 0.614 | 0.545 |
| EG669717 | Rc234 | AATCCAGAGAGGCAAAACATTC | CACCATTGAAAGCCAGCTACTA | (TA)_12_ | 238 | 232-248 | 6 | 0.768 | 0.734 |
| EE260480 | Rc236 | TTGGTTGGAAAAACAGATTCCT | AAACCCGCTTGAAATATGATTG | (CT)_12_ | 250 | 250-258 | 2 | 0.486 | 0.368 |
| EG676447 | Rc239 | CCAACAAATTCACTCCCTCTCT | CCAAGATTGAAGCAAAAACACA | (TC)_13_ | 203 | 209-223 | 3 | 0.531 | 0.428 |
| EG695527 | Rc242 | ACCCCTGCAAAACCCTAATAAT | TTCGGTGTAAAGAATCCGACTT | (CT)_13_ | 243 | 243-259 | 3 | 0.469 | 0.391 |
| EE254642 | Rc243 | CAGAAGAAGCAGTAGCAGCAAA | CCCAAGTCCCAGATCTCTCTAA | (TA)_14_ | 203 | 196-228 | 4 | 0.503 | 0.456 |
| EG669444 | Rc245 | TCACTTTTACCTCCCTCTGCTC | ATGGGTTTAGGAATGTTGTTGG | (CT)_14_ | 230 | 230-249 | 3 | 0.538 | 0.480 |
| EE259040 | Rc246 | TGCAGTTTCGTCTCCTTCACTA | AATGACAATGGCGACTGATAAA | (AG)_14_ | 193 | 193-196 | 2 | 0.375 | 0.305 |
| EG680607 | Rc247 | AACAAAATCCAAACCCCAATC | CAGCATATGATTGTTCCTTCCA | (AT)_15_ | 229 | 229-247 | 3 | 0.258 | 0.240 |
|  |  |  |  |  |  |  |  |  |  |
| EG681668 | Rc248 | CAATCACCACCAAAAACCAAAG | CAGCATATGATTGTTCCTTCCA | (AT)_15_ | 213 | 213-233 | 4 | 0.263 | 0.252 |
| EG683395 | Rc249 | GACTTCAACTTTCTACCTGACCTTG | CTACCACCTGATTTCCCCATAA | (CT)_15_ | 226 | 226-245 | 3 | 0.288 | 0.264 |
| EG661770 | Rc251 | AAACAAACAACACCGAAATCCT | CGTAGCGACTCATTTACACCAA | (CT)_15_ | 187 | 181-195 | 4 | 0.605 | 0.526 |
| EG667814 | Rc256 | AGACAAACACATGCTGCCTTTA | ATGTGTGGTGGGGATGTTATCT | (AGAA)_5_ | 186 | 181-188 | 3 | 0.635 | 0.561 |
| EG662055 | Rc258 | GCAGTTAGGCATCAAAATCCTC | GAAGTGCAGCTGTTAGGGTTTC | (GAAA)_5_ | 201 | 194-202 | 3 | 0.655 | 0.581 |
| EG692953 | Rc259 | ACGACTCCGACTCCTCTTACAC | TTTCTTCTTTAATCGGCGTCTC | (AAAG)_5_ | 191 | 193-205 | 2 | 0.153 | 0.141 |
| EG658641 | Rc260 | GAAGTGCAGCTGTTAGGGTTTC | GCAGTTAGGCATCAAAATCCTC | (TTTC)_5_ | 201 | 201-209 | 2 | 0.457 | 0.353 |
| EG664709 | Rc262 | ACGACTCCGACTCCTCTTACAC | TTTCTTCTTTAATCGGCGTCTC | (AAAG)_5_ | 191 | 191-199 | 2 | 0.153 | 0.141 |
| EV523864 | Rc272 | ACGATCTGCTTGTTCTCTGT | CAGGTCAAGATCTCCAACAT | (GCG)_4_ | 238 | 235-246 | 4 | 0.370 | 0.327 |
| EV523770 | Rc287 | CCTAAGGCAGCTAAGTCAAA | GCAACTGCTTATGCTTCTCT | (CTG)_5_ | 217 | 312-327 | 3 | 0.492 | 0.435 |
| EV523693 | Rc294 | GAATCCCAATGTCCAGTCTA | CCTGAAAGAGAACAAAGTGG | (TCT)_5_ | 155 | 152-161 | 3 | 0.469 | 0.391 |
| EV523589 | Rc317 | CGCCAAGCTTATAAGAGAAA | GAGTCTGAAGGGAGAGGAAT | (AAG)_4_ | 149 | 149-161 | 3 | 0.223 | 0.206 |
| EV523460 | Rc325 | ATCACCCACAATTACCCATA | CGAGAGAACGAAAAGAGAAA | (TC)_5_ | 132 | 130-136 | 2 | 0.080 | 0.077 |
| EV523432 | Rc328 | GCAGAAAGAACACGAATCTC | AGCAATAAAAACCAAGCAAG | (TC)_12_ | 107 | 105-123 | 5 | 0.654 | 0.596 |
| EV523389 | Rc331 | TTAGCTGCTTCGATTTCTTC | CAACATTAGCAGGCTGTGTA | (GAG)_4_ | 208 | 208-216 | 2 | 0.413 | 0.328 |
| EV521100 | Rc357 | ATGGAGTTTGAGGATCAAGA | AACTCGCTGTCATTTTGACT | (GAA)_6_ | 121 | 122-125 | 2 | 0.219 | 0.195 |
| EV521100 | Rc358 | AGTCAAAATGACAGCGAGTT | CGTGGTTCTTTAAGCACTCT | (CAG)_6_ | 119 | 121-130 | 2 | 0.278 | 0.239 |
| EV520933 | Rc365 | CCATCGCTTGTCTAGTGTTT | CATTACCCAAAGAAGACTCG | (TAA)_6_ | 144 | 144-151 | 3 | 0.452 | 0.381 |
| EV520791 | Rc374 | GATCTCGTAATGAATGTTGG | CTCTCTTCTTGAGGCTCTTC | (GCT)_5_ | 143 | 266-272 | 3 | 0.312 | 0.274 |
|  |  |  |  |  |  | Average | 2.97 | 0.414 | 0.361 |
|  |  |  |  |  |  |  |  |  |  |
