# Supplementary material for: Exploiting EST databases for the development and characterization of EST-SSR markers in castor bean (Ricinus communis L.)
Source: BMC Plant Biol. 2010 Dec 16;10:278. doi: 10.1186/1471-2229-10-278 (PMC3017068; doi:10.1186/1471-2229-10-278)
Supplement: Additional file 3 — Table S3: Homology with Aradidopsis and functional annotations of the EST-SSR markers.doc [file 1471-2229-10-278-S3.DOC]

**Table S3: Homology with *Aradidopsis* and functional annotations of the EST-SSR markers**

| **Code** | **Genbank ID** | **Homology in *Arabidopsis*** | **Annotation** | **e-Value** | **SSR position** |
| --- | --- | --- | --- | --- | --- |
| RC02 | EG662973 | AT3G06590 | Transcription factor | 5e-08 | 5’ UTR |
| RC03 | EG686206 | -* |  |  | 5’ UTR |
| RC04 | EG682908 | AT5G51300 | Splicing factor-related; RNA binding zinc ion and nucleotide binding | 4e-59 | 5’ UTR |
| RC05 | EE255832 | At1g44170 | Putative aldehyde dehydrogenase | 3e-74 | 5’ UTR |
| RC07 | EG663122 | AT2G40960 | Nucleic acid binding | 3e-62 | 5’ UTR |
| RC09 | EG686478 | AT2G28950 | ATEXPA6 | 5e-90 | 5’ UTR |
| RC10 | EG678906 | - |  |  | 5’ UTR |
| RC11 | GE634375 | AT5G13190 | Unknown protein | 8e-20 | 5’ UTR |
| RC12 | T14845 | - |  |  | UTR |
| RC14 | EG666688 | - |  |  | 5’ UTR |
| RC16 | EE259292 | - |  |  | 5’ UTR |
| RC17 | EG674726 | - |  |  | 5’ UTR |
| RC19 | EG670576 | AT2G46170 | Reticulon family protein (RTNLB5) | 6e-13 | 3’ UTR |
| RC20 | EE259262 | AT2G25737 | Unknown protein | 2e-31 | 5’ UTR |
| RC22 | EE256592 | - |  |  | 3’ UTR |
| RC23 | EG662293 | AT2G39940 | COI1; ubiquitin-protein ligase | 3e-11 | 5’ UTR |
| RC24 | EE255894 | AT5G17165 | Unknown protein | 3e-26 | 3’ UTR |
| RC25 | GE633816 | AT1G48970 | GTP binding / translation initiation factor | 9e-19 | 5’ UTR |
| RC26 | EG664176 | AT1G48330 | Unknown protein | 6e-04 | 3’ UTR |
| RC27 | EG684662 | AT1G33470 | RNA recognition motif-containing protein; RNA binding, nucleotide binding, nucleic acid binding | 5e-50 | 5’ UTR |
| RC28 | EE260610 | AT5G56170 | Unknown protein | 2e-42 | 5’ UTR |
| RC29 | EE257519 | - |  |  | UTR |
| RC31 | GE632365 | - |  |  | CDs |
| RC32 | EE254685 | - |  |  | UTR |
| RC33 | GE632805 | - |  |  | 3’ UTR |
| RC35 | EE260187 | AT3G27809 | Unknown protein | 2e-04 | 3’ UTR |
| RC40 | EE254189 | - |  |  | 3’ UTR |
| RC45 | EG698508 | AT1G07310 | C2 domain-containing protein | 2e-23 | CDs |
| RC51 | EG664367 | AT1G07310 | C2 domain-containing protein | 6e-39 | CDs |
| RC53 | EG697581 | AT3G54390 | Transcription factor | 8e-55 | CDs |
| RC57 | EG689318 | - |  |  | CDs |
| RC61 | EG662586 | AT2G37220 | RNA-binding protein cp29, putative; RNA binding, poly(U) binding | 2e-73 | CDs |
| RC65 | EG701975 | AT2G28380 | DRB2; double-stranded RNA binding | 2e-101 | 5’ UTR |
| RC73 | GE633761 | - |  |  | CDs |
| RC74 | EG698249 | AT1G06760 | Histone H1, putative; DNA binding | 3e-19 | CDs |
| RC76 | EG696213 | - |  |  | 5’ UTR |
| RC84 | EG695475 | AT5G67230 | Glycosyl transferase family 43 protein; 3-beta-glucuronosyltrans- ferase activity, transferase activity, transferring glycosyl groups | 2e-67 | 5’ UTR |
| RC85 | EG660729 | - |  |  | CDs |
| RC86 | EG681190 | AT4G32551 | LUG (LEUNIG); protein binding /protein heterodimerization/ transcription repressor | 1e-48 | CDs |
| RC87 | EG687367 | - |  |  | 5’ UTR |
| RC88 | EG667050 | AT3G46740 | TOC75-III; P-P-bond-hydrolysis-driven protein transmembrane transporter | 2e-61 | CDs |
| RC89 | EG679278 | - |  |  | 3’ UTR |
| RC90 | EG666840 | AT1G70420 | Unknown protein | 7e-39 | CDs |
| RC91 | EG665468 | AT3G53630 | Unknown protein | 4e-58 | CDs |
| RC94 | EG691818 | AT1G68050 | FKF1; signal transducer/ two-component sensor/ ubiquitin-protein ligase | 4e-87 | CDs |
| RC95 | EG699176 | AT3G06130 | Heavy-metal-associated domain-containing protein; metal ion binding | 2e-31 | CDs |
| RC96 | EG693205 | - |  |  | 5’ UTR |
| RC100 | EG681785 | AT2G21350 | RNA binding | 1e-18 | CDs |
| RT103 | EG678636 | - |  |  | 5’ UTR |
| RT104 | EG686206 | - |  |  | 5’ UTR |
| RT112 | EE254243 | - |  |  | UTR |
| RT113 | EG679802 | AT4G20260 | PCAP1; phosphatidylinositol-3,4,5-triphosphate binding, copper ion binding, calmodulin binding, phosphatidylinositol-3,5- bisphosphate binding, calcium ion binding | 1e-12 | 3’ UTR |
| RT116 | EG689244 | - |  |  | 5’ UTR |
| RT117 | EG691558 | - |  |  | 5’ UTR |
| RT119 | EG663853 | AT5G54110 | ATMAMI; structural molecule activity | 2e-54 | 5’ UTR |
| RT121 | EG697485 | AT1G07090 | LSH6 | 2e-31 | 5’ UTR |
| RT122 | EG669444 | - |  |  | 5’ UTR |
| RT124 | EE256105 | AT1G75390 | AtbZIP44; transcription factor activity, protein heterodimerization activity, DNA binding | 2e-16 | 3’ UTR |
| RT126 | GE632416 | AT2G44760 | Unknown protein | 6e-71 | 5’ UTR |
| RT128 | EG689641 | AT2G40830 | RHC1A; protein binding / zinc ion binding | 2e-17 | 5’ UTR |
| RT129 | GE634251 | AT1G32740 | Protein binding / zinc ion binding | 2e-26 | 5’ UTR |
| RT130 | EG692156 | AT1G33800 | Unknown protein | 6e-64 | 5’ UTR |
| RT131 | EG685152 | - |  |  | UTR |
| RT132 | EG694164 | AT5G14040 | Mitochondrial phosphate transporter | 1e-83 | 5’ UTR |
| RT135 | GE634416 | AT5G21280 | Hydroxyproline-rich glycoprotein family protein | 3e-16 | 5’ UTR |
| RT138 | EG680179 | - |  |  | 5’ UTR |
| RT139 | EE254161 | AT5G17165 | Unknown protein | 2e-23 | 3’ UTR |
| RT140 | EE259204 | - |  |  | UTR |
| RT141 | EG700448 | AT4G34640 | SQS1; farnesyl-diphosphate farnesyltransferase | 1e-61 | 5’ UTR |
| RT158 | EG693883 | AT4G02440 | EID1; ubiquitin-protein ligase activity | 3e-40 | CDs |
| RT165 | GE635881 | AT5G58530 | Glutaredoxin family protein; electron carrier activity, protein disulfide oxidoreductase activity | 3e-36 | CDs |
| RT166 | EG684973 | - |  |  | CDs |
| RT167 | GE632520 | AT1G12520 | ATCCS; superoxide dismutase copper chaperone activity, superoxide dismutase activity | 5e-78 | CDs |
| RT168 | GE633169 | AT5G65780 | ATBCAT-5; branched-chain-amino-acid transaminase activity | 2e-64 | 5’ UTR |
| RT176 | GE633829 | AT1G23780 | F-box family protein | 2e-18 | CDs |
| RT182 | GE635438 | AT4G17040 | ATP-dependent Clp protease proteolytic subunit, putative; serine-type endopeptidase activity | 2e-90 | CDs |
| RT184 | EG671871 | - |  |  | UTR |
| RT186 | EG683300 | AT1G63480 | DNA-binding family protein | 8e-05 | CDs |
| RT188 | EG662044 | AT1G07080 | GILT family protein; catalytic activity | 3e-64 | 5’ UTR |
| RT189 | EG666688 | - |  |  | 5’ UTR |
| RT193 | EE258383 | AT1G23710 | Unknown protein | 1e-34 | CDs |
| RT194 | EG662514 | AT1G71480 | NTF2 family protein; protein transporter activity | 7e-30 | 5’ UTR |
| RT195 | EG673660 | AT1G13450 | DNA binding protein GT-1; transcription factor activity | 2e-16 | 3’ UTR |
| RT197 | EG667076 | - |  |  | 5’ UTR |
| RT206 | GE634515 | AT2G46700 | CDPK, putative | 1e-21 | 5’ UTR |
| RT220 | EE259493 | AT1G32740 | Protein binding /zinc ion binding | 6e-04 | CDs |
| RT223 | EE255709 | AT2G28840 | Ankyrin repeat family protein; protein binding, zinc ion binding | 1e-11 | 3’ UTR |
| RT224 | EG695020 | - |  |  | UTR |
| RT226 | EE254893 | AT2G18280 | AtTLP2; phosphoric diester hydrolase/transcription factor | 2e-33 | CDs |
| RT228 | EE254441 | - |  |  | 5’ UTR |
| RT233 | EG657403 | AT4G14550 | Indole-3-acetic acid inducible 14 | 7e-52 | 5’ UTR |
| RT234 | EG669717 | - |  |  | 5’ UTR |
| RT236 | EE260480 | - |  |  | 3’ UTR |
| RT239 | EG676447 | - |  |  | UTR |
| RT242 | EG695527 | AT1G48970 | GTP binding /translation initiation factor | 3e-18 | 5’ UTR |
| RT243 | EE254642 | - |  |  | 3’ UTR |
| RT245 | EG669444 | - |  |  | 5’ UTR |
| RT246 | EE259040 | AT3G04040 | Unknown protein | 4e-47 | 3’ UTR |
| RT247 | EG680607 | - |  |  | 5’ UTR |
| RT248 | EG681668 | - |  |  | 3’ UTR |
| RT249 | EG683395 | AT1G62390 | Octicosapeptide/Phox/Bem1p domain-containing protein / tetratricopeptide repeat (TPR)-containing protein | 2e-66 | 5’ UTR |
| RT251 | EG661770 | AT2G20840 | SCAMP family protein; transmembrane transporter activity | 8e-72 | 5’ UTR |
| RT256 | EG667814 | AT1G78310 | VQ motif-containing protein | 1e-27 | 5’ UTR |
| RT258 | EG662055 | - |  |  | 5’ UTR |
| RT259 | EG692953 | AT3G58970 | Magnesium transporter CorA-like family protein; metal ion transmembrane transporter activity | 3e-45 | 5’ UTR |
| RT260 | EG658641 | AT2G45190 | AFO; protein binding / transcription factor/transcription regulator | 3e-61 | 3’ UTR |
| RT262 | EG664709 | AT3G58970 | Magnesium transporter CorA-like family protein; metal ion transmembrane transporter activity | 6e-40 | 5’ UTR |
| RC272 | EV523864 | AT4G34110 | PAB2; RNA binding /translation initiation factor | 7e-79 | CDs |
| RC287 | EV523770 | AT2G30620 | Histone H1.2; DNA binding | 1e-27 | CDs |
| RC294 | EV523693 | AT5G49980 | Auxin F-Box protein 5; ubiquitin-protein ligase | 1e-44 | 5’ UTR |
| RC317 | EV523589 | AT2G25910 | 3’-5’ exonuclease domain-containing protein /Khomology domain-containing protein/KH domain-containing protein; 3’-5’ exonuclease activity, RNA binding, nucleic acid binding | 1e-95 | 5’ UTR |
| RC325 | EV523460 | AT2G37035 | Unknown protein | 4e-21 | 5’ UTR |
| RC328 | EV523432 | AT3G02470 | SAMDC; adenosylmethioninedecarboxylase | 7e-37 | 5’ UTR |
| RC331 | EV523389 | AT4G30920 | Cytosol aminopeptidase family protein; manganese ion binding, metalloexopeptidase activity, aminopeptidase activity | 9e-94 | CDs |
| RC357 | EV521100 | AT4G24660 | ATHB22; transcription factor activity, DNA binding | 6e-22 | CDs |
| RC358 | EV521100 | AT4G24660 | ATHB22; DNA binding /transcription factor | 6e-22 | CDs |
| RC365 | EV520933 | AT5G51210 | OLEOSIN3 | 3e-28 | 3’ UTR |
| RC374 | EV520791 | AT5G24510 | 60s acidic ribosomal protein P1, putative; structural constituent of ribosome | 3e-31 | CDs |

- No homology was identified in *Arabidopsis*
